# Tumor Infiltrating Lymphocyte Therapy Combined With PD‐1/LAG‐3 Inhibition in Patients With Recurrent Platinum‐Resistant Ovarian Cancer

**DOI:** 10.1002/ijc.70510

**Published:** 2026-04-23

**Authors:** Tine J. Monberg, Cathrine L. Lorentzen, Marie C. W. Westergaard, Trine Z. Iversen, Troels H. Borch, Marco Donia, Sigrid M. Mannering, Stine E. W. Banke, Özcan Met, Inge Marie Svane

**Affiliations:** ^1^ National Center for Cancer Immune Therapy (CCIT‐DK), Department of Oncology Copenhagen University Hospital Herlev Denmark; ^2^ Department of Oncology Copenhagen University Hospital Herlev Denmark; ^3^ Department of Health Technology The Technical University of Denmark (DTU) Kgs. Lyngby Denmark

**Keywords:** immune checkpoint inhibitors, lymphocyte activating gene 3, ovarian cancer, tumor infiltrating lymphocyte therapy

## Abstract

Patients with ovarian cancer (OC) have not yet benefitted from the advances in immuno‐oncology. While tumor infiltrating lymphocytes (TILs) can be expanded from OC tumors, previous trials have not demonstrated lasting responses. High expression of the immune checkpoints PD‐1 and LAG‐3 on TILs from OC provide a rationale for the addition of immune checkpoint inhibitors (ICI) to the treatment. In this clinical pilot study (NCT04611126), five patients with platinum‐resistant recurrent OC were treated with TIL therapy and up to four cycles of combined treatment with PD‐1‐/LAG‐3‐inhibitors. The primary endpoint was safety and feasibility, while secondary endpoints included immune monitoring and clinical efficacy. Included patients had undifferentiated carcinoma (*n* = 1), high‐grade serous OC (HGSOC) (*n* = 2) and low‐grade serous OC (LGSOC) (*n* = 2). The treatment was safe and feasible with expected treatment‐related toxicity; however, there was a relatively high rate of non‐treatment‐related complications. A decrease in tumor burden was observed in 80% (4/5) of patients, including two unconfirmed partial responses. In one patient, the response was supported by in vitro reactivity of the infused TILs toward autologous tumor cell line. Differences in baseline tumor burden, infusion product composition and responses were observed in LGSOC vs. HGSOC. Overall, this exploratory pilot study demonstrated a favorable safety profile and indications of clinical efficacy for TIL therapy combined with PD‐1 and LAG‐3 inhibition in platinum‐resistant OC. Due to the low patient number, the results should be interpreted as hypothesis‐generating, providing a rationale for conducting larger trials that carefully consider treatment timing and tumor histology.

## Abstract (highlights)

What's new?

The detection of tumor‐infiltrating lymphocytes (TILs) is a positive prognostic factor in ovarian cancer. Moreover, TILs are significantly boosted by immunotherapy, though ovarian cancer patients have seen limited benefit from immune therapies. This study investigated the safety and feasibility of TIL therapy combined with PD‐1 and LAG‐3 inhibitors in five patients with platinum‐resistant recurrent ovarian cancer. Treatment was feasible, with expected toxicity. Tumor burden decreased in 80% of patients, with variation by tumor subtype. The findings indicate that combined TIL and checkpoint inhibition is a valid clinical strategy for ovarian cancer, supporting further investigation in trials with more patients.

## Graphical abstract

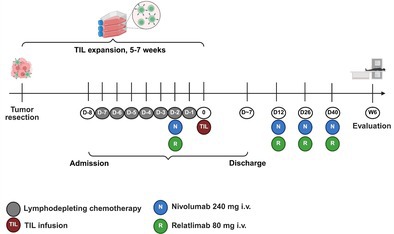

**Abbreviations**
ACT: adoptive cell therapy
AE: adverse event
BOR: best overall response
CA‐125: cancer antigen 125
CM: culturing media
CMV: cytomegalovirus
dMMR: mismatch repair deficient
ECOG PS: Eastern Cooperative Oncology Group performance status
EM: effector memory
HDR: homology‐directed repair
HGSOC: high‐grade serous ovarian cancer
HRD: homologous recombination deficiency
ICI: immune checkpoint inhibitors
IFNg: interferon gamma
Ki‐67: antigen Kiel 67
LAG‐3: lymphocyte activating gene 3
LGSOC: low‐grade serous ovarian cancer
LVEF: left ventricular ejection fraction
MHC: major histocompatibility complex
MSI‐H: microsatellite instability‐high
OC: ovarian cancer
ORR: overall response rate
OS: overall survival
PARP: poly (ADP‐ribose) polymerase
PD: progressive disease
PD‐1: programmed cell death protein 1
PFS: progression‐free survival
PR: partial response
RECIST: response evaluation criteria in solid tumors
REP: rapid expansion protocol
RR: response rate
SAR: serious adverse reaction
SD: stable disease
TD: tumor digest
TIL: tumor‐infiltrating lymphocyte
TMB: tumor mutational burden
TNF: tumor necrosis factor

## 1 Introduction

Ovarian Cancer (OC) is the 17th most common cancer in women and has the highest mortality rate among gynecological cancers [1]. OC is a heterogenous group of malignancies, largely defined as tumors originating from the ovaries, the fallopian tubes, or the peritoneal wall. However, the definition of OC varies across the world and has changed over time [2]. The most common type of OC is the epithelial carcinomas comprising ~90% of all OC cases, but also within this subgroup the heterogeneity is high, and the prognosis varies significantly [3]. High‐grade serous ovarian cancer (HGSOC) is the most frequent type comprising ~80% of the epithelial OCs. Other subtypes include clear cell carcinoma, endometrioid cancer, mucinous carcinoma, and low‐grade serous ovarian cancer (LGSOC); however, all at relatively low frequencies [2]. Despite the high heterogeneity, epithelial OCs are treated according to the same standards. Initial treatment includes surgery combined with (adjuvant or neo‐adjuvant) platinum‐based chemotherapy. Efficacy is high, especially in HGSOC, but ~70% of patients will experience recurrence within 3 years [4]. Within the last decade, targeted therapies with anti‐angiogenesis drugs and poly (ADP‐ribose) polymerase (PARP) inhibitors alone or in combination have been approved as maintenance therapy after first‐line chemotherapy [5, 6]. These drugs significantly improve progression‐free survival (PFS), particularly in patients with BRCA1 or BRCA2 mutations (somatic or germline) or homologous recombination deficiency (HRD) [7, 8, 9]. However, the overall 5‐year survival remains below 50% and has not improved noteworthy within the last decade [10, 11]. Further, for the 15% of patients with primary platinum‐resistant or ‐refractory disease, the prognosis is very poor [7, 8].

The immunogenicity of OC has been debated, but as in most other cancers, the presence of tumor infiltrating lymphocytes (TILs) is a good prognostic factor [12]. However, while the use of immune checkpoint inhibitors (ICI) is rapidly expanding to several cancer types, no immunotherapeutic agent has been approved for the treatment of OC and the efficacy of ICI monotherapy in OC has so far been disappointing [13, 14]. Combination therapy with ipilimumab and nivolumab was reported to result in a higher response rate (RR) and significantly longer, but still very limited, PFS compared with nivolumab monotherapy in platinum‐resistant OC patients [15]. Importantly, convincing evidence that immunotherapy performs better than chemotherapy in platinum‐resistant OC is still awaited.

Similarly, treatment with TIL therapy is a poorly explored area in OC. Preclinical work at our center found that TILs can be successfully expanded from OC tumor tissue; however, the number of tumor reactive T‐cells was found to be limited [16]. In a meta‐analysis of adoptive cell therapy (ACT) in gynecological cancers, a RR of 22.6% in OC was reported [17], but RRs for TIL therapy alone were not described. Selective expansion of tumor reactive TILs or genetically engineered T cells represents promising strategies to improve ACT in OC, but evidence of efficacy is still lacking [18]. We have previously conducted two clinical pilot studies demonstrating that TIL therapy is safe and feasible in patients with platinum‐resistant HGSOC [19, 20]. A high expression of the checkpoint inhibitors programmed cell death protein 1 (PD‐1) and lymphocyte activating gene 3 (LAG‐3) on the infused TILs was found in most patients; this, along with a high expression of PD‐L1 and MHC class II on the tumor cells, indicated that the addition of ICI to the treatment regimen could possibly increase efficacy [19, 20]. Indeed, with the addition of a PD‐1‐inhibitor, short‐lived partial responses (PRs) were obtained in two patients [20].

Here, we report the results from a clinical pilot study including five patients with platinum‐resistant, recurrent, epithelial OC treated with TIL therapy plus up to four cycles of combined treatment with the PD‐1 inhibitor nivolumab and the LAG‐3 inhibitor relatlimab.

## 2 Materials and Methods

### 2.1 Trial Design

The trial was conducted as a phase I/II clinical study. The original protocol aimed to enroll 18 patients, divided into three cohorts of six patients each. However, the study was terminated prematurely due to challenges in patient recruitment, and the planned sample size was therefore not achieved.

All patients were treated at the National Center for Cancer Immune Therapy (CCIT‐DK) and the Department of Oncology, Copenhagen University Hospital, Herlev, Denmark. Criteria for inclusion and exclusion are shown in Paragraph S1. Eligible patients had platinum‐resistant or ‐refractory metastatic OC with no restriction on numbers of previous lines of therapy, an Eastern Cooperative Oncology Group (ECOG) performance status (PS) of 0–1, acceptable organ function and a metastasis of at least 1 cm^3^ available for surgical resection. Further, all patients underwent an echocardiographic assessment of the left ventricular ejection fraction (LVEF) and only patients with a LVEF ≥ 50% were included in the trial.

**FIGURE 1:**
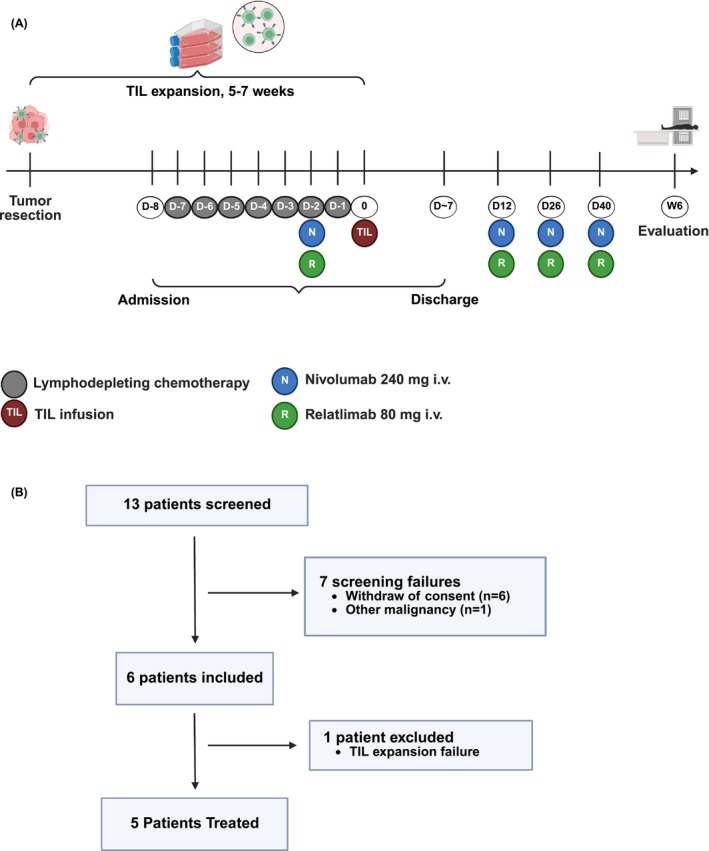
(A) Treatment schedule. The total TIL expansion time was 5–7 weeks. Prior to TIL infusion, the patient was admitted for 7 days of lymphodepleting chemotherapy with cyclophosphamide and fludarabine phosphate. The first dose of nivolumab/relatlimab was administered 2 days before TIL infusion. The remaining three doses of nivolumab/relatlimab were administered every 2nd week for a maximum of four cycles. The initial assessment of efficacy was performed 6 weeks post TIL infusion. TIL, tumor infiltrating lymphocytes. (B) Trial CONSORT diagram. Of the 13 patients screened, 6 patients were included in the protocol, and 5 patients received TIL therapy. Created with biorender.com.

All included patients followed the treatment schedule shown in Figure 1A. Approximately 6 weeks after tumor resection, the patient was admitted for TIL therapy preceded by 7 days of lymphodepleting chemotherapy with cyclophosphamide (60 mg/kg for 2 days) followed by fludarabine phosphate (25 mg/m^2^ for 5 days), as previously described [20]. Combined treatment with nivolumab (BMS) 240 mg and relatlimab (BMS) 80 mg was administered 2 days before TIL infusion. Nivolumab/relatlimab infusions were repeated biweekly for a maximum of 4 cycles. The first evaluation of response was performed 6 weeks post TIL therapy.

### 2.2 TIL Generation

The workflow for TIL generation and reactivity testing is outlined in Figure S1. Tumor resection was, with one exception, performed at the Department of Gynecology and Obstetrics, Copenhagen University Hospital, Herlev, Denmark. Resected tumor tissue was placed in a sterile container with RPMI‐1640 (Gibco, Thermo‐Fisher) and 1% Penstrep (Penicillin/Streptomycin, Gibco) and transported to Good Manufacturing Practice (GMP) clean room facility for dissection into fragments of 1–3 mm^3^. Young TILs [21] from the fragments were expanded for 3–5 weeks in a culturing media (CM) containing RPMI‐1640 (Gibco, Thermo‐Fisher), 10% heat‐inactivated human AB serum (Sigma Aldrich), 1% Penstrep (penicillin/streptomycin, Gibco), 0.5% Fungizone (BMS) and 6000 IU/mL interleukin‐2 (Proleukin, Novartis). The cells were then expanded in accordance with the rapid expansion protocol (REP) as previously described [22]. In one patient young TILs were cryopreserved for later REP expansion and treatment. The REP was initiated in static culture containers with 2 × 10^7^ TILs in cell culture medium containing RPMI 1640/AIM‐V (Thermo Fisher Scientific), 10% human serum, IL‐2, anti‐CD3‐antibody (OKT3) (Miltenyi Biotec) and irradiated allogeneic feeder cells in a 1:200 ratio. Between Day 6 and 9 of the REP, depending on cell growth, the cell culture was transferred to a dynamic Xuri W25 bioreactor (Cytiva). On Day 14, the cell culture was harvested, centrifuged, and washed prior to patient infusion.

### 2.3 Phenotypic Characterization of Infusion Products

Young TILs and REP TILs were phenotypically characterized by flow cytometry using the three panels presented in Table S1. In short, cryopreserved samples of young TILs and infusion products were thawed in pulmozyme buffer serum, plated in CM, and washed twice. Subsequently, the cells were stained and incubated for 30 min at 4°C in the dark. The cells were analyzed on a Novocyte Quanteon Flow Cytometer with Novoexpress software (Agilent).

### 2.4 Reactivity Testing by Intracellular Staining

The tumor reactivity of young TILs and REP TILs was tested toward autologous tumor cell lines (TCL) or tumor digest (TD). Tumor cells from fragments or transport media were expanded in RPMI‐1640 (Gibco, Thermo‐Fisher), 10% fetal bovine serum (ThermoFisher), 1% Penstrep (penicillin/streptomycin, Gibco), 1% sodium pyruvate (Sigma‐Aldrich (Merck)), 0.5% Fungizone (BMS) and 0.1% insulin (Sigma‐Aldrich (Merck)). TD was generated from resected tumor tissue through enzymatic digestion (BD dissociation kit, BD 661563).

Tumor cells were stimulated with 100 IU/mL IFNg (BD Biosciences) for 72 h, whereafter TILs and tumor cells were co‐cultured 3:1 (E:T ratio) for 8 h with anti‐human CD107a antibody, brefeldin A (GolgiPlug, dilution of 1:1000), and monensin (GolgiStop, dilution 1:1000). After the 8 h of incubation, the cells were washed in DPBS and stained with live/dead (NIR) and surface markers (CD3, CD8, CD4, CD137, CD107a). Cells were then fixed and permeabilized for 1 h, followed by a wash and staining for intracellular markers (tumor necrosis factor [TNF] and interferon gamma [IFNg]). The cells were analyzed on a Novocyte Quanteon Flow Cytometer with NovoExpress software (Agilent). As previously reported, cells positive for at least two markers (CD137, TNF, CD107a and/or IFNg) were considered tumor reactive [23].

### 2.5 Data Collection and Analysis

Study data were collected and managed using REDCap electronic data capture tools hosted at Region Hovedstaden [24, 25]. All data analyses were performed in RStudio version 4.3.2 using the packages ggplot2 [26], ggsurvfit [27] and tidyverse [28].

### 2.6 Outcomes

The primary objective of this study was to evaluate the safety of the treatment using the National Cancer Institute (NCI) Common Terminology Criteria for Adverse Events (CTCAE) version 5.0 [29]. Secondary objectives included immune profiling with phenotypic characterization of infusion products and assessment of antitumor immune responses. Further, assessment of clinical efficacy evaluated by RECIST 1.1, cancer antigen 125 (CA‐125) measurements, and assessment of overall survival (OS) and PFS is described.

The data cut off was the 1st of June 2025.

## 3 Results

### 3.1 Patient Population

Six patients with platinum‐resistant metastatic OC were included in the trial from April 2021 to September 2023. The CONSORT diagram is presented in Figure 1B. Patient recruitment was very slow, mainly due to low referral of patients meeting the eligibility criteria, and the trial was closed prematurely for this reason. Thirteen patients were screened, but seven were not included due to either patient's wish (*n* = 6) or the occurrence of another malignancy (*n* = 1). Further, of the six included patients, one patient (04) with LGSOC was excluded due to unsuccessful TIL expansion. Baseline characteristics of the remaining five patients are shown in Table 1. Histologically, the patients differed markedly, including one patient with undifferentiated carcinoma (01), two patients with LGSOC (02 and 03) and two patients with HGSOC (05 and 06). All patients were in a good ECOG PS of 0–1 at the time of inclusion. Median age was 62 years (range 45–70), and patients had received a median of three prior treatment lines (range 3–4). The time span from the primary diagnoses to TIL treatment varied from 9 to 60 months (median 26 months). Further, the burden of disease at baseline varied significantly with three patients (01, 05, 06) having organ involvement and disease spread above the diaphragm muscle and two patients (02, 03) having more localized disease in the abdomen. Similarly, the aggressiveness of the disease, as illustrated by the pathological markers listed in Table 1 (antigen Kiel 67 (KI‐67), number of mitoses, tumor mutational burden (TMB) or homology‐directed repair [HDR]), differed between patients.

**TABLE 1:** Baseline characteristics of the five treated patients.

Baseline characteristics											
Patient	Age	Time to TIL	Histology	PS	No. of treatment lines	Prior treatment lines	Sites of disease	CA‐125 (kU/L)	BRCA	ER	Aggressiveness
01	45	9	Undifferentiated carcinoma	1	3	Carboplatin/Paclitaxel, Caelyx/Bevacizumab Topotecan	Parasternal, intraabdominal, lymph nodes in inguen, subcutaneous	17	No	Negative	> 10 mitosis per 10 HPF
02	47	26	Low‐grade serous adenocarcinoma	0	4	Carboplatin/Paclitaxel/Bevacizumab, Carboplatin/Paclitaxel, Carboplatin/Doxorubicin/Letrozol, Carboplatin/Caelyx	Intraabdominal, carcinomatosis	130	No	Positive	TMB low
03	67	60	Low‐grade serous adenocarcinoma	0	3	Carboplatin/Paclitaxel/Letrozol (A) Carboplatin/Doxorubicin/Letrozol (A) Carboplatin/Paclitaxel (A)	Carcinomatosis	16	No	Positive	KI‐67 10%–30%, TMB low
05	70	21	High‐grade serous adenocarcinoma	0	3	Carboplatin/Paclitaxel (NA) + PARP‐I, Doxorubicin/Bevacizumab, Bevacizumab/Tocotrienol (Ex)	Pleura, intraabdominal, retroperitoneal, liver	21,700	BRCA1	Positive	KI‐67 50%, HDR‐negative
06	62	28	High‐grade serous adenocarcinoma	1	3	Carboplatin/Paclitaxel (NA), PIPAC Doxorubicin	Carcinomatosis, intraabdominal, mediastinum, peritoneum, lymph nodes on neck and in axilla, inguinal lymph nodes, pleura	500	No	Unknown	KI‐67 20%–30%, HDR negative
Median	62	26	—	0	3	—	—	130	—	—	—

*Note:* Patients differed histologically with both low‐grade serous adenocarcinoma (*n* = 2), high‐grade serous adenocarcinoma (*n* = 2), and undifferentiated carcinoma (*n* = 1) being represented. All patients were in a good Eastern Cooperative Oncology Group (ECOG) performance status (PS). The median age was 62 years (range 45–70). Patients were heavily pretreated (median of 3 prior treatment lines, range 3–4). The time span from the primary diagnoses to TIL treatment varied from 9 to 60 months (median 26 months). Three patients (01, 05, 06) had disease spread above the diaphragm muscle. The aggressiveness of the disease is illustrated by the pathological markers: Antigen Kiel 67 (KI‐67), number of mitoses, tumor mutational burden (TMB), and homology‐directed repair (HDR). Aggressiveness = pathological markers of cancer aggressiveness.

Abbreviations: A, adjuvant; BRCA, somatic mutations in BRCA1 or BRCA2; ER, estrogen receptor; Ex, experimental; HDR, homology‐directed repair; HPF, high‐power field; NA, neoadjuvant; PIPAC, pressurized intraperitoneal aerosol chemotherapy; PS, performance status; Time to TIL, months from diagnosis to TIL treatment; TMB, tumor mutational burden.

Patient 01 was diagnosed with a highly aggressive, undifferentiated carcinoma originating from the ovaries, characterized by a high mitotic rate. The patient received 3 lines of chemotherapy prior to TIL therapy but progressed clinically through all lines of treatment before the planned radiological evaluations. Patients 02 and 03, both diagnosed with LGSOC, had slowly progressing disease with low TMB and limited disease burden when enrolled in the trial. Patient 05 with HGSOC had a highly aggressive cancer with a CA‐125 level of 21.700 kU/L at inclusion and a KI‐67 value of 50%. Finally, Patient 06 had widespread disease and accelerated disease progression with the appearance of numerous pleural metastases prior to TIL treatment.

### 3.2 Safety and Feasibility

All treatment related adverse event (AE) are shown in Table 2A while serious adverse reactions (SARs) are shown in Table 2B.

**TABLE 2:** Toxicity of the treatment. (A) All treatment‐related adverse events (AEs) registered in the trial. *n* = number of patients. All patients developed Grade 4 neutropenia and lymphopenia related to the chemotherapy. Few grade ≥ 3 AEs were treatment‐related. PS, performance status. (B) Serious adverse reactions (SARs) occurred in three patients, two of which were related to the treatment with immune checkpoint inhibitors (ICI). (C) Treatment characteristics including hematological toxicity are shown as days with severe neutropenia (median 6, range 4–8) and the number of platelet (median 2, range 0–3) and erythrocyte (median 2, range 0–6) transfusions.

(A) Treatment‐related adverse events		
	Any grade, *n* (%)	Grade ≥ 3, *n* (%)
**Chemotherapy**		
Anemia	5 (100%)	1 (20%)
Hyponatremia	5 (100%)	1 (20%)
Lymphopenia	5 (100%)	5 (100%)
Nausea	5 (100%)	0
Neutropenia	5 (100%)	5 (100%)
Thrombocytopenia	5 (100%)	4 (80%)
Decrease in PS	4 (80%)	1 (20%)
Fatigue	4 (80%)	1 (20%)
Infections	3 (60%)	2 (40%)
Vomiting	3 (60%)	0
Diarrhea	2 (40%)	0
Obstipation	2 (40%)	0
Dry mouth	1 (20%)	0
Dyspnea	1 (20%)	0
Elevated ALAT/ASAT	1 (20%)	0
Increased p‐ferritin	1 (20%)	1 (20%)
Oral candida	1 (20%)	0
**TIL product**		
Fever	4 (80%)	0
Fatigue	3 (60%)	0
Decrease in PS	2 (40%)	0
Elevated ALAT/ASAT	1 (20%)	0
Maculopapular rash	1 (20%)	1 (20%)
**Checkpoint inhibitors**		
Hyperthyroidism	2 (40%)	0
Maculopapular rash	2 (40%)	1 (20%)
Dry mouth	1 (20%)	0
Dyspnea	1 (20%)	0
Fever	1 (20%)	0
Pain	1 (20%)	0
Troponin increase	1 (20%)	0

(B) Serious adverse reactions (SARs)				
Patient	SAR	Suspected drug/intervention	Action	Consequence
02	Hyperthyroidism	ICI	Antithyroid drug therapy	Treatment with ICI discontinued
05	Bacterial infection during neutropenia	Chemotherapy	Antibitotics	NA
06	CMV reactivation	Chemotherapy	Antiviral medication	Treatment with ICI discontinued

(C) Hematological toxicity					
Patient	Days of grade 4 neutropenia	Number of platelet transfusions	Number of erythrocyte transfusions	Nivolumab/relatlimab doses	Admissions duration (days)
01	4	0	4	3	20
02	8	2	2	3	18
03	6	0	0	4	15
05	7	3	6	2	20
06	6	3	2	3	20
Median	6	2	2	3	20

Abbreviation: ICI, immune checkpoint inhibitors.

No complications were registered in relation to the surgery. All included patients developed anemia, hyponatremia, lymphopenia, nausea, neutropenia, and thrombocytopenia following chemotherapy (Table 2A). Further, most patients experienced a decrease in PS (80%), fatigue (80%), infections (60%), and vomiting (60%). The median duration of Grade 4 neutropenia was 6 days (range 4–8) (Table 2C). Three out of five (60%) of patients needed platelet transfusion (transfusion limit = platelets < 20 × 10^9^/L) and 4/5 (80%) patients received erythrocyte transfusion during the admission (transfusion limit = hemoglobin < 6 mmol/L) (Table 2C). Other severe (≥ grade 3) chemotherapy‐related AEs included infections (40%), decreased PS (20%), fatigue (20%), and increased p‐ferritin (20%) (Table 2A).

The most frequently reported AEs related to the TIL infusion were fever (80%) and fatigue (60%) (Table 2A). One patient (05) developed Grade 3 maculopapular rash on Day 6 and 7 following TIL infusion, possibly related to either TIL‐infusion, nivolumab/relatlimab, or the combination of these drugs. The rash resolved spontaneously without any medical interventions.

Overall, the treatment with nivolumab/relatlimab was well‐tolerated with few treatment‐related AEs. One patient (02) developed Grade 2 hyperthyroidism following the third dose of nivolumab/relatlimab. Symptoms (tachycardia, diarrhea, dyspnea, and tremor) did not resolve upon standard treatment with beta receptor blocking and the patient was further treated with prednisolone and propylthiouracil on which all symptoms relieved (Table 2B). The patient did not receive the last dose of nivolumab/relatlimab. Further, Patient 06 did not receive the 4th dose of nivolumab/relatlimab due to a severe reactivation of latent cytomegalovirus (CMV), which was attributed to the lymphodepleting chemotherapy (Table 2B).

Other reasons for early discontinuation of ICI (Patients 01 and 05) were clinical deterioration and/or disease progression. The median number of nivolumab/relatlimab cycles was 3 (Table 2C).

Additional significant AEs included two cases of fatal ileus (Patients 01 and 05) 36 and 63 days after TIL infusion, respectively. In both patients imaging revealed a transition point with proximal bowel dilatation, but no definitive mechanical obstruction was identified, and the underlying cause remained unclear. In Patient 01 the transition point was localized in the small intestine, while Patient 05 had an obstruction in the colon. Both patients had carcinosis in the affected area. Patient 05 underwent successful colon stenting but did not improve clinically. Further, the clinical course of Patient 05 was complicated by an active and severe *clostridium difficile* infection, which might have contributed to the development of ileus ultimately leading to shock and fatal renal failure. Due to the severity of the condition both patients were treated with corticosteroids, but experienced rapid clinical deterioration shortly after admission, precluding gut biopsy.

### 3.3 Clinical Efficacy

**FIGURE 2:**
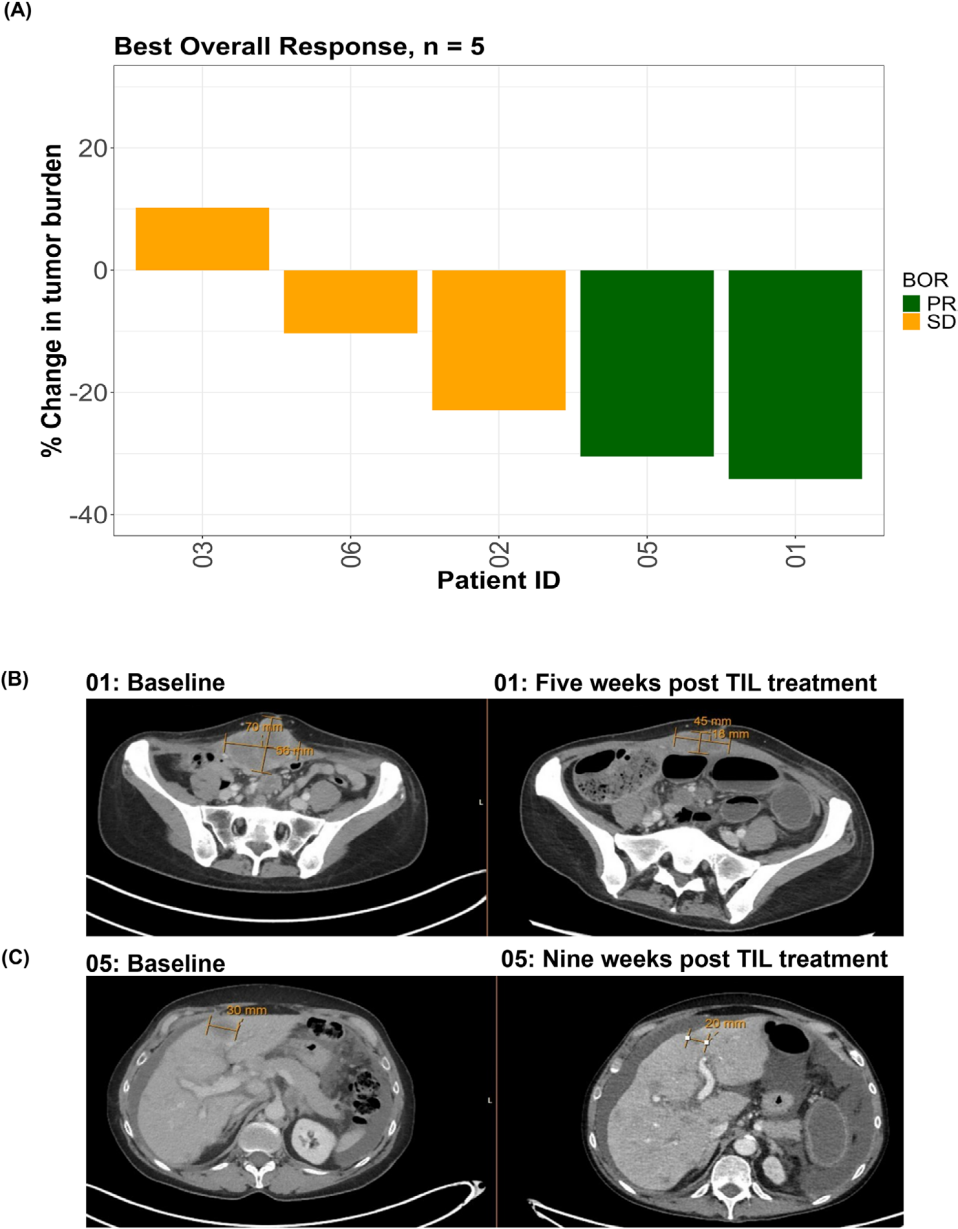
Efficacy of the treatment. (A) Best overall response (BOR) presented as the best percentage chance in tumor burden compared to baseline. PR, partial response; SD, stable disease. Partial responses were unconfirmed. (B and C) CT scans from the two patients (01 and 05) that developed an unconfirmed PR. Patient 01 developed a remarkable reduction at all sites, in particular in a metastasis in the peritoneal wall (B). Similarly, Patient 05 developed tumor reduction in most sites including numerous liver metastases (C).

Treatment efficacy is shown in Figure 2. In 4/5 (80%) of patients, a reduction in tumor burden (best overall response [BOR]) was observed (Figure 2A). In two patients (01 and 05), the BOR met the criteria of a PR (Figure 2B,C). Patient 01 had a high tumor burden when included in the trial and, due to rapid progression, the patient needed local radiotherapy of a peritoneal wall tumor in the waiting time for TIL therapy. During admission, the patient received only one dose of cyclophosphamide due to low tolerability. The patient experienced clinical improvement after discharge but developed a fatal case of ileus 5 weeks post TIL treatment. Notably, a PR with a 34% decrease in the size of target lesions was observed at the time of ileus development (Figure 2A,B). However, this response could not be confirmed because of the early death of the patient.

Patient 05 showed a decrease in CA‐125 (29%) (Figure S2A) and target lesions (19%) (Figure S2B) 6 weeks post TIL treatment, but unfortunately, the patient died from colon ileus 9 weeks post TIL treatment. An evaluation performed at the time of the bowel obstruction revealed a PR with a 30.4% decrease in the size of target lesions (Figure 2C).

Two patients (02 and 03) had stable disease (SD) (Figure 2A). On the first scan Patient 02 had a reduction in tumor burden (−23%) as well as CA125 (−63%) (Figure S2), while Patient 03 had a slight increase (+10%) in tumor burden and a doubling of CA‐125 (Figures 2A and S2). From 6 weeks post TIL treatment, both patients had a steady increase in tumor burden until fulfilling the criteria for progression, respectively 5.3‐ and 9‐months post therapy.

Patient (06) progressed within 3 months from TIL treatment. The first evaluation 6 weeks post treatment showed a 10% decrease in tumor burden. However, due to metastases localized to the pericardium, the patient developed life‐threatening pericardial effusion 9 weeks post treatment. Symptoms were relieved by pericardial drainage, but a CT scan performed 12 weeks post treatment confirmed PD with the appearance of one new metastasis.

The median PFS was 92 days (Figure S3A), while median OS was 116 days (Figure S3B).

At data cut off, one patient (02) is still alive, 1278 days post TIL treatment.

### 3.4 Expansion Characteristics and Phenotypic Traits of Infused TILs

**FIGURE 3:**
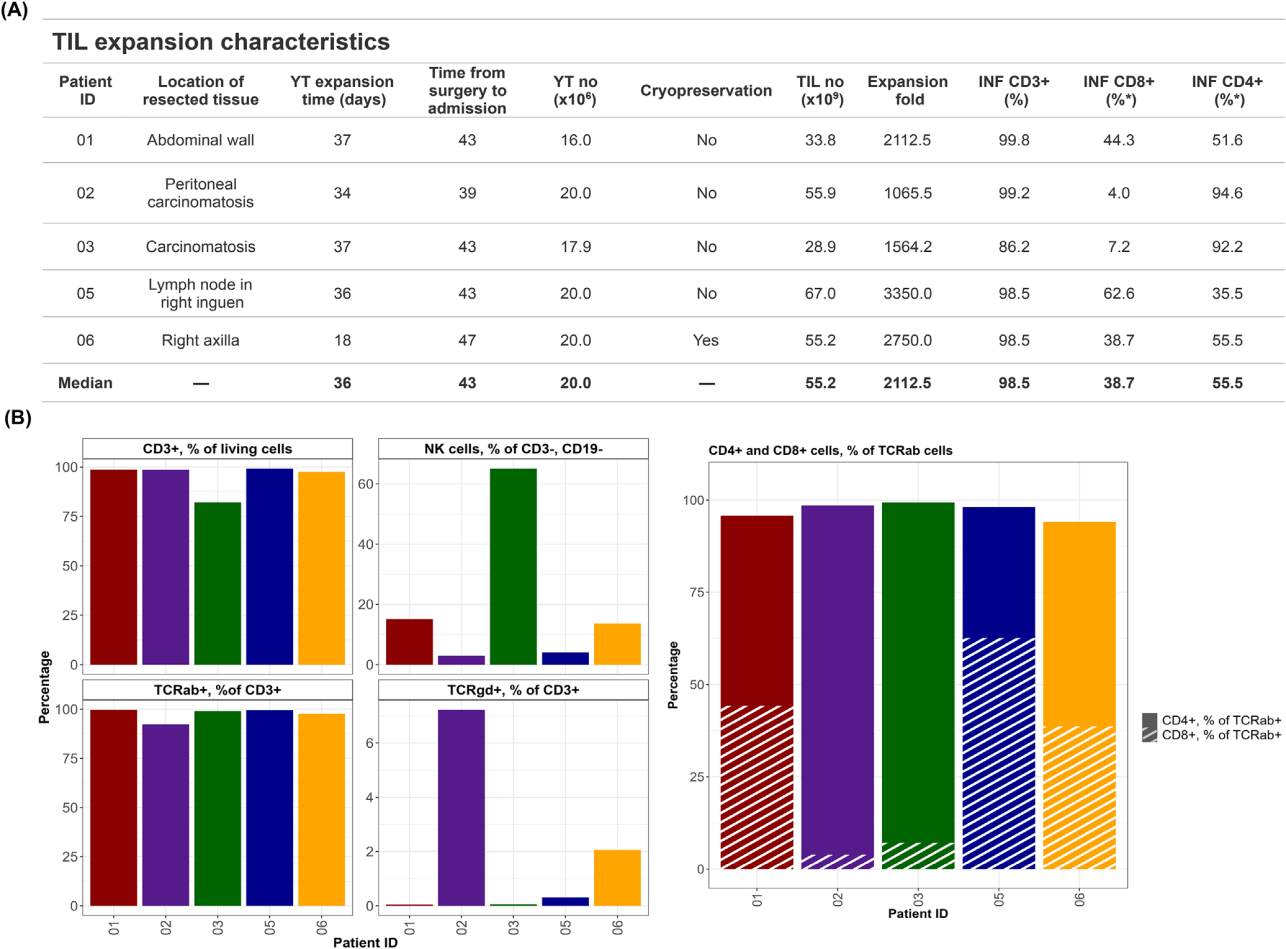
TIL expansion characteristics. (A) Individual TIL growing characteristics. The young TIL (YT) expansion times ranged from 18 to 37 days. In two patients the number of YT entering the rapid expansion protocol (REP) was lower than the intended number of 20 × 10^6^. The total number of infused TILs ranged from 28.9 × 10^9^ to 67.0 × 10^9^. * = of TCRab (B) Distribution of different cell subsets in the infusion product of each patient. For all patients the TIL infusion product consisted almost exclusively of CD3+ cells, but the distribution of CD4+ and CD8+ T cells varied significantly. Patients diagnosed with low‐grade serous ovarian cancer (LGSOC) tended to have infusion products primarily dominated by CD4+ T cells. NK, natural killer cells; TCRab, T‐cell receptor alpha beta; TCRgd, T‐cell receptor gamma delta.

Individual TIL expansion characteristics are shown in Figure 3A. The median young TIL expansion time was 36 days (18–37 days) which is longer than previously reported in OC [20]. Due to slow growth the number of young TILs entering the REP was below 20 × 10^6^ cells in two patients (01 and 03) (median 20 × 10^6^, range: 16–20 × 10^6^). The median number of infused cells was 55 × 10^9^ (range 29–67 × 10^9^) cells.

The infusion products consisted almost exclusively of CD3+ cells (median 98%, range: 82%–99% of live singlet cells) (Figure 3B). However, in one patient (03), 18% of the cells were CD3‐CD19‐, and a considerable fraction (65%) of these were positive for CD56+ consistent with a population of natural killer (NK) cells (Figure 3B). Most of the CD3 cells were TCR alpha‐beta (TCRαβ) positive (Figure 3B). The median fraction of CD4+ cells was 56% (range 36%–95%), while the median fraction of CD8+ cells was 39% (range 4%–63%) (Figure 3B). The one patient with undifferentiated carcinoma (01) and the two patients with HGSOC (05 and 06) all had relatively high fractions of CD8+ T cells (44%, 63%, and 39% of CD3+ TCRαβ cells, respectively). In the two patients with LGSOC (02 and 03), the infusion products consisted almost exclusively of CD4+ T‐cells (95% and 92% of CD3+ TCRαβ, respectively).

**FIGURE 4:**
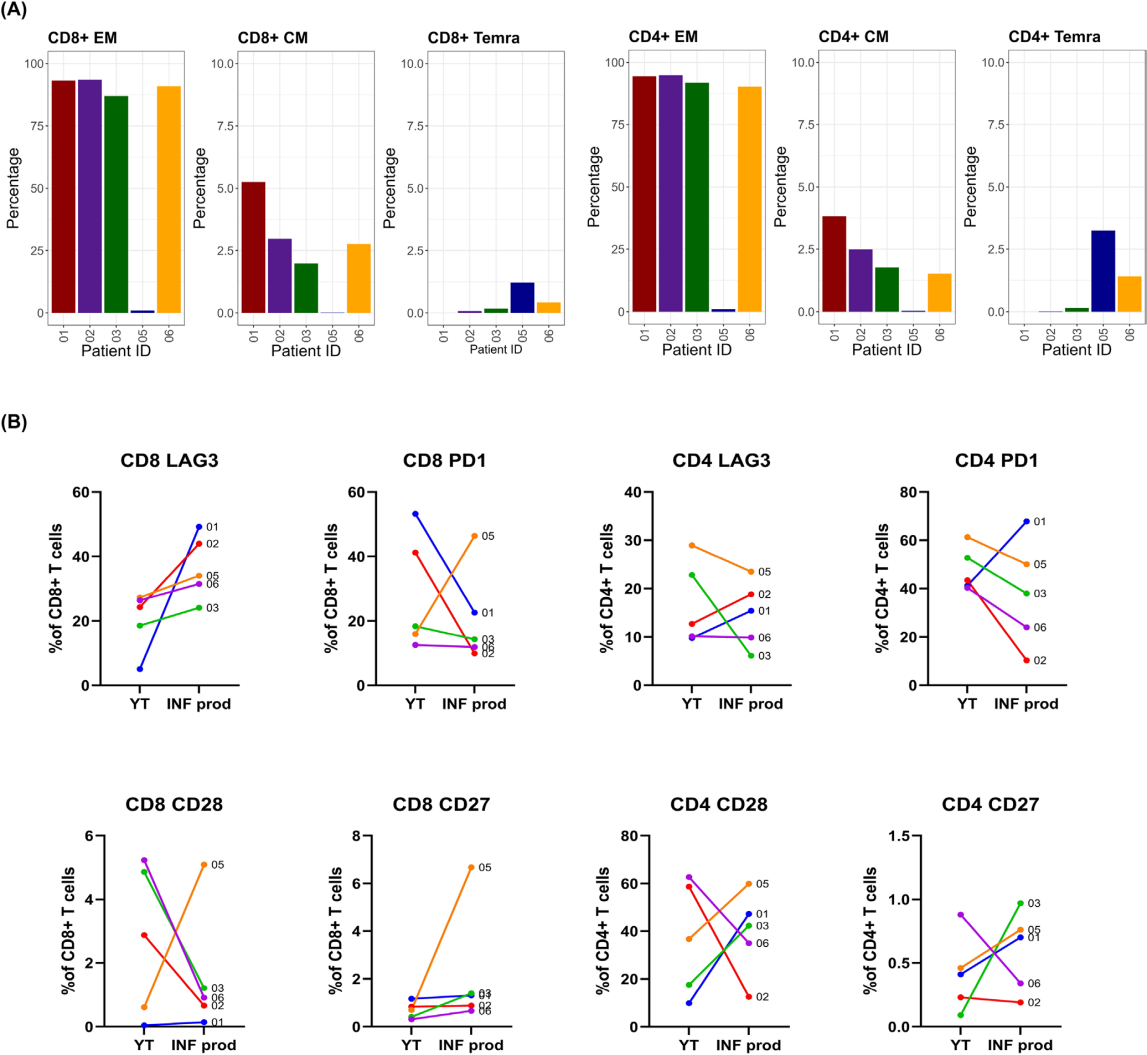
Flow cytometry‐based phenotyping of infusion products. (A) Different cell populations within the CD8+ and CD4+ subsets for each patient. CM, central memory; EM, effector memory; TEMRA, terminally differentiated memory T‐cells. In Patient 05, most of the cells could not be phenotypically characterized; see Figure S5. (B) Changes in the expression of selected markers during the REP. In TIL infusion products, LAG3 expression was highest on CD8+ T cells (median 34%, range 24%–49%), whereas PD‐1 expression was highest on CD4+ T cells (median 38%, range 10%–67%). No clear trend was observed. LAG3, lymphocyte activation gene 3. PD‐1, programmed cell death protein 1.

The infusion products were further characterized using staining panel 2 and 3 (Table S1). In 4 of 5 patients, the expanded TILs could almost exclusively be characterized as effector memory (EM) cells (CD45RO+, CCR7‐) (Figures 4A and S4). However, Patient 01 had a considerable increase in the fraction of central memory (CM) CD8+ T cells (1%–5%) during the REP (Figure S4). Further, in one patient (05), most of the cells from both young TILs and Rep TILs were double positive for CD45RA and CD45RO but lacked expression of CCR7 (Figures 4A and S5). These cells could not be further characterized according to the established understanding of T cell development [30].

Changes in LAG‐3 and PD‐1 expression during the REP is illustrated in Figure 4B. The median percentage of CD8+ and CD4+ cells in the infusion products expressing LAG‐3 was 34% (range 24%–49%) and 15.4% (range 6%–23%), respectively. PD‐1 expression was most prominent on CD4+ cells with a median of 38% (range 10%–67%), whereas CD8+ cells showed a median PD‐1 expression of 14% (range 10%–46%).

The expression of the costimulatory markers CD27 and CD28 declined in CD8+T‐cells during the REP in most patients (Figure 4B). Of note is, however, Patient 05 in which the expression of these markers increased on both CD4+ and CD8+ cell subsets.

The expression of selected markers (Panel 3, Table S1) is shown in Figure S6. In most patients, a high expression of CD39 and a low expression of CD103 were detected on both CD4+ and CD8+ cell subsets. No other clear tendencies were observed.

### 3.5 In Vitro Reactivity of Infusion Products Toward Autologous Tumor Tissue

Autologous tumor cell lines were attempted generated in three patients (01, 02, and 06). To verify the presence of tumor cells, immunohistochemical analysis was conducted, which revealed that the cells could be characterized as tumor cells only in one patient (01). TD was available for reactivity testing from Patient 01, 02, 05, and 06.

In Patient 01 antitumor reactivity was detected after stimulation of tumor cells with IFNg (Figure S7A), with an observed increase in the fraction of TNF/IFNg double‐positive CD8+ T cells from 0% to 40%. Similarly, CD137 expression on CD8+ cells increased from 1.3% to 60% when tested toward IFNg treated tumor cells. A similar tendency was observed when post‐treatment (Day 12) CD8+ PBMCs from this patient were tested toward IFNg treated tumor cells (Figure S7A). To explore this finding further, tumor cells from Patient 01 were stained for the expression of MHC I and MHC II before and after stimulation with IFNg. We observed a 21‐fold increase in the expression of MHC I after stimulation with IFNg, while MCH II expression remained almost unaffected (Figure S7B). PD‐L1 expression was retrospectively assessed from a diagnostic tumor sample, showing a PD‐L1 expression (tumor proportion score) of 15%–20%.

In Patients 02 and 05, we observed no clear indication of reactivity (data from Patient 05 shown in Figure S8A).

In Patient 06, reactivity with expression of several markers was observed in the CD4+ T cell population when tested against TD, while the expression of reactivity markers was low in the CD8+ T cell population (Figure S8B).

## 4 Discussion

Recurrent epithelial OC remains a diagnosis with a poor prognosis. The approval of PARP inhibitors has significantly increased the platinum‐free PFS, especially in HGSOC with germline BCRA1/2 mutations or somatic BRCA mutations/HRD [9]. However, at the time of platinum‐resistant relapse, available treatment options have very little efficacy. Convincing efficacy of immunotherapy is still pending, and the immunogenicity of OC is continuously debated [31].

Accounting for the small sample size, the current trial indicates that TIL therapy can be safely combined with PD‐1 and LAG‐3 checkpoint inhibition in patients with OC. Toxicity related to the TIL infusion and the lymphodepleting chemotherapy was expected and manageable, and the median duration of Grade 4 neutropenia (6 days) was comparable with our previous findings in OC [20]. One patient developed a severe reactivation of CMV 3 weeks post TIL infusion. While CMV reactivation has not previously been reported in relation to TIL therapy, it is a relatively common complication related to the lymphodepletion in CAR‐T therapy [32]. However, severe CMV reactivation with the need for antiviral therapy is rare and associated with poor survival [32].

AEs related to the treatment with ICI were manageable and comparable with those observed in melanoma patients receiving nivolumab/relatlimab [33]. Also, the main reason for early discontinuation of the ICI treatment was clinical deterioration with no relation to the ICI treatment itself.

Tumor shrinkage was observed in 80% (4/5) of the treated patients. However, in the two patients who obtained a PR, follow‐up assessments to confirm responses were not possible. The poor outcomes for these two patients were attributed to the underlying malignancy, not the treatment itself. Bowel obstruction is a common phenomenon in patients with intraabdominal late‐stage malignant diseases, with a reported incidence as high as 51% in OC [34, 35]. Its causes are multifactorial, and the prognosis is generally poor, with only a few selected patients benefitting from surgery [34]. However, bowel obstruction is generally described in the context of cancer progression or recurrence [34], thus usually not associated with tumor regression. Nevertheless, considering the intra‐abdominal tumor burden in both patients, the development of ileus is a known risk, and the two cases of bowel obstruction emphasize the fact that the included patients had end‐stage disease and a very poor prognosis. However, although the malignant disease itself likely played a major role, therapy‐related factors cannot be completely excluded. The high‐dose chemotherapy regimen may have disturbed the intestinal balance, thereby increasing vulnerability. This consideration is particularly relevant for Patient 05, which had an active *clostridium difficile* infection, which is a well‐known cause of toxic megacolon and ileus [36]. ICI‐related ileus is a rare phenomenon, primarily described in isolated case reports and usually occurring in association with ICI‐induced colitis rather than as an isolated event [37]. Taken together, although possibly associated with the high‐dose chemotherapy, the most plausible explanations for the development of ileus in the two cases appear to be infection, tumor‐related changes, and/or high intra‐abdominal disease burden, rather than a direct effect of ICI therapy.

Due to the histopathological differences among the patients in this trial, inter‐patient comparison was not meaningful; however, a few overall tendencies were seen. In general, the trial population was heavily pretreated with a median of 3, primarily chemo‐based, prior treatment lines. The effect of chemotherapy on response to later immunotherapy is unclear, but the responsiveness of most cancers declines with the number of prior therapies [38].

Further, 3/5 patients had highly aggressive disease with accelerated progression and increasing CA‐125 (in 2/3 patients) in the waiting time for TIL therapy. Consequently, the tumor burden was high, and the disease had become symptomatic at the time of admission. In melanoma, disease burden as well as LDH level is known to be negatively associated with response to immunotherapy [39]. Similar data are not available for OC, but most likely the same is true. In conclusion, it cannot be ruled out that the fragile patient population in this trial influenced outcomes negatively.

Systemic immunological changes following lymphodepleting chemotherapy, TILs and anti‐PD‐1 therapy have previously been described in a larger cohort (*n* = 31) of patients with solid tumors, including OC [40]. The treatment induces dramatic changes within the circulating lymphocyte compartment, including a rapid decline in CD4+ T cells within the first 6 weeks after therapy. CD8+ T cells are not affected to the same degree, which might reflect the high proportion of CD8+ T cells within many (though not all) infusion products. Furthermore, naïve and central memory T cells almost disappear from the circulation and these cells are still affected 6 months after treatment [40]. In contrast, EM cells tend to increase, possibly reflecting the high proportion of these cells in TIL infusion products. Interestingly, PD‐1 expression on circulating T cells appears to decline over time in patients receiving anti‐PD‐1 therapy in combination with TILs and chemotherapy. These systemic effects on peripheral immune cells have been documented across different tumor types [40]. Regarding functional parameters, studies in melanoma have shown that persistence of tumor‐reactive CD8+ T cells correlates with durable clinical responses [41, 42, 43]. These cells can be characterized by their distinct phenotype and cytokine profile upon stimulation with tumor cells ex vivo [43]. However, a prognostic profile based on cytokine measurements in peripheral blood has not been established.

Because of the small sample size and short follow‐up in the current study, we did not assess changes in systemic immune functions following therapy. Including these measures in future OC TIL trials will provide a better understanding of changes in the host immune after therapy and how these changes might affect clinical outcome.

The results from the phenotyping of infusion products were comparable to previous findings on TIL infusion products from both OC [20] and other cancer types [44] showing mostly effector memory T cells with high expression of checkpoint and/or markers of exhaustion [19, 20].

In responder Patient 01 we observed a remarkably high proportion of tumor‐reactive CD8+ T cells in the infusion product when tested toward IFNg stimulated autologous tumor cells. IFNg was found to dramatically increase the expression of MHC I on tumor cells, thereby changing the range of the displayed peptides [45]. This, possibly, explains the drastic change in reactivity. Considering the aggressiveness of the disease and resistance to prior lines of chemotherapy, it seems unlikely that Patient 01 could have benefitted from the high dose chemotherapy alone. Further, this patient had a relatively high expression of LAG‐3 (49%) and PD‐1 (22%) on infused CD8+ TILs and the tumor tissue was PD‐L1 positive. Overall, the results suggest that the treatment mediated the tumor regression in this patient.

In Patient 05, who also developed an unconfirmed PR, we saw a co‐expression of CD45RA and CD45RO combined with no expression of CCR7 on almost all infused TILs. Although these cells displayed an interesting “young” phenotype with high expression of CD27 and CD28, they have not been described previously and we were not able to demonstrate antitumor reactivity. Thus, the functional significance of these cells remains unclear.

In Patients 02 and 03, the overall disease development seemed to be following the natural course of LGSOC with slow but steady progression. In general, TIL infiltration of LGSOC tumors is reported to be low and, in contrast to HGSOC, no association with OS has been reported [46]. The absence of in vitro reactivity of the TILs (Patient 02) combined with the phenotyping results indicate that the expanded TILs in these two patients did not possess tumor killing potential. The transient reduction in tumor burden in Patient 02 was most likely mediated by the high dose chemotherapy.

Direct comparison with our previous clinical trials testing TIL therapy in patients with OC [19, 20] is challenged by the omission of postconditioning IL‐2 in the current trial.

Still, compared to our previous trials we found that relatively fewer of the infused CD4+ and CD8+ TILs expressed PD‐1 and LAG‐3 [19, 20]. Also, there was no clear tendency toward increased expression of these markers after the REP. These phenotypic results might, however, be influenced by the use of different clonotypes of the LAG‐3 antibody. In general, despite lower expression than previously reported, the expression of LAG‐3 (median 34% of CD8+ T cells) on infused TILs was high enough to provide a rationale for the use of relatlimab. However, it must be noted that expression of PD‐1, PD‐L1 and LAG‐3 does not necessarily reflect the functional activity of these immune checkpoints as post‐translational modifications (phosphorylation or ubiquitination) are required for full activation [47]. This underscores that expression alone is insufficient for identifying patients that are most likely to benefit from TIL therapy combined with ICI treatment, and it highlights that development of additional, clinically applicable functional tests is required.

Two major limitations of the current trial are the small sample size and histological heterogeneity of the patient population. OC is a highly diverse disease and the findings from this study cannot be generalized to other histological subtypes. This underscores the need to account for OC heterogeneity when designing future OC studies. Most literature on TILs in OC concerns HGSOC as this is the most frequent subtype. Encouraging results, with an overall response rate (ORR) of 33%, were recently reported for the combination of nivolumab and ipilimumab in a subgroup of OC patients with gynecological clear cell carcinomas [48]. These results confirm that the histology of OC influences the success of immunotherapeutic treatment [49]. This was further supported in a prospective study which found that higher levels of tumoral CD8+ T cell infiltration are associated with increased survival in HGSOC. Interestingly, this relation could not be shown in LGSOC [46]. In addition, evidence support that OC with Microsatellite Instability‐High (MSI‐H) or mismatch repair deficient (dMMR) OC might benefit more from PD‐1 blockade, presumably due to the higher neoantigen load, and thereby a higher TIL infiltration, in these tumors [50].

Histological subtypes with lower immunogenicity and limited baseline TIL infiltration (etc. LGSOC) constitute hard‐to‐treat patient populations. In these patients, strategies to increase baseline T cell infiltration are needed. Overall, careful patient selection to identify patients which will most likely benefit from TIL treatment is highly important in future OC TIL trials.

The full potential of TILs + ICI could not be elucidated in the current trial. The recruitment rate was unexpectedly slow, leading to an early discontinuation of the trial. The widespread use of PARP inhibitors for HGSOC, as well as the approval of MEK inhibitors for LGSOC, might have affected the recruitment rate. Further, dropout during the screening period was high (*n* = 6). The primary factor contributing to the withdrawal of consent was that the patients felt physically and psychologically unable to go through the treatment.

Responding patients died from malignancy associated complications, which implies that TIL therapy was introduced too late. Most OC patients undergo debulking surgery at the time of diagnosis. Given that most of these patients will relapse within few years [4] the surgery provides an opportunity to harvest, expand and cryopreserve TILs for future use. Interestingly, a recent report indicates that neoadjuvant chemotherapy in OC alters the tumor microenvironment in a way that might enhance TIL infiltration or immune response [51]. Further, in a recent clinical trial, platinum‐based chemotherapy was used for lymphodepletion before TIL therapy in patients with recurrent platinum‐sensitive OC. The ORR was 86%, which is comparable with platinum‐chemotherapy. However, in one patient the platinum‐free interval was > 42 months compared to 8 months on prior platinum‐based chemotherapy alone in this patient [52]. Based on these results, an appealing strategy could be to harvest, expand and cryopreserve TILs during the initial debulking surgery. The optimal timing for TIL therapy remains undefined, but the likelihood of response may decrease with an increasing number of prior treatment lines. For clinical trial design, TIL therapy could therefore be considered in combination with platinum‐based chemotherapy at relapse or as first‐line treatment after the development of platinum‐resistant disease.

In conclusion, TIL therapy combined with PD‐1/LAG‐3 inhibition was a safe and feasible treatment modality with early indications of clinical efficacy in this small cohort of patients with platinum‐resistant OC. However, given the small sample size and the histological heterogeneity of the patient population, no clear conclusions can be drawn. This study should therefore be considered hypothesis‐generating, and it highlights the need for larger stratified trials.

To unveil the full potential of TIL therapy in OC, the timing of the treatment as well as upfront genetic profiling, including BRCA/HRD and TMB/immunoscoring, should be assessed. Further, the heterogeneity of the disease should be considered in future clinical trials.

## Author Contributions

**Tine J. Monberg:** investigation, visualization, writing – review and editing, writing – original draft, formal analysis. **Cathrine L. Lorentzen:** investigation, writing – review and editing. **Marie C. W. Westergaard:** investigation, visualization, writing – review and editing, formal analysis. **Trine Z. Iversen:** investigation, writing – review and editing. **Troels H. Borch:** investigation, writing – review and editing. **Marco Donia:** writing – review and editing. **Sigrid M. Mannering:** investigation, writing – review and editing. **Stine E. W. Banke:** investigation, writing – review and editing. **Özcan Met:** investigation, writing – review and editing. **Inge Marie Svane:** conceptualization, funding acquisition, writing – review and editing, supervision, project administration, resources, investigation.

## Funding

This study was initiated and sponsored by the National Center for Cancer Immune Therapy (CCIT‑DK) in cooperation with the Department of Oncology, University Hospital Herlev, Denmark, and was conducted using institutional resources from these departments. Additional expenses were covered from a research grant from the private funding organization OvaCure. Bristol Meyers Squibb (BMS) provided relatlimab for free for the treatment of patients included in the trial.

## Ethics Statement

The trial is registered on clinicaltrials.gov, NCT04611126. Before enrolment in the trial, all patients provided both written and oral informed consent in accordance with the declaration of Helsinki. The trial was approved by the Danish National Ethics Committee (approval no. 2008209) and the Danish Medicines Agency. All approvals were obtained prior to inclusion of the first patient in the trial. The trial was monitored by The Good Clinical Practice (GCP) unit, Frederiksberg, Denmark.

## Conflicts of Interest

T.H.B. has received personal payment for lectures/presentation from Bristol Myers Squibb (BMS) and from MSD. M.D. has received advisory fees from Achilles Therapeutics, and consultancy fees via membership of Guidepoint LLC and AlphaSights expert network. I.M.S.: Personal payments honoraria: received for lectures, presentations, speakers' bureaus, manuscript writing, advisory board or educational events from MSD, Takeda, Sanofi Aventis, Janssen Cilag and BMS, Institutional grants and contracts: received from Evaxion Biotech, Adaptimmune, IO Biotech, Asgard Biotech, TILT Biotherapeutics and Enara Bio, Consulting fees: received from TILT Biotherapeutics, IO Biotech, Novartis and Genmab, Stocks/shares: IO Biotech, Meeting support: received from MSD, Clinical trial drugs: Received Relatlimab from BMS, DSMB participation: involved in only academic trials. The remaining authors declare no conflicts of interest.

## Supporting information


**Paragraph S1:** Inclusion and exclusion criteria.
**Figure S1:** TIL expansion and reactivity testing workflow.
**Figure S2:** Individual changes in CA‐125 and tumor burden during treatment.
**Figure S3:** Treatment efficacy.
**Figure S4:** Changes in populations of CD8+ and CD4+ cells during the REP.
**Figure S5:** Phenotyping of young TILs and REP TILs in Patient 05.
**Figure S6:** Flow‐cytometry based phenotyping of TIL infusion products.
**Figure S7:** Tumor reactivity of TILs from Patient 01.
**Figure S8:** Tumor reactivity of TILs from Patients 05 and 06.
**Table S1:** Staining panels used for flow‐cytometry based phenotyping of TIL infusion products.

## Data Availability

De‐identified data may be made available upon reasonable request to the corresponding author, subject to institutional and ethical approvals. Further information that supports the findings of this study is available from the corresponding author.
