# Supplementary material for: Tumor Infiltrating Lymphocyte Therapy Combined With PD‐1/LAG‐3 Inhibition in Patients With Recurrent Platinum‐Resistant Ovarian Cancer
Source: Int J Cancer. 2026 Apr 23;159(7):1735–49. doi: 10.1002/ijc.70510 (PMC13432351; doi:10.1002/ijc.70510)
Supplement: Supplementary file 1 — Paragraph S1: Inclusion and exclusion criteria. Figure S1: TIL expansion and reactivity testing workflow. Figure S2: Individual changes in CA‐125 and tumor burden during treatment. Figure S3: Treatment efficacy. Figure S4: Changes in populations of CD8+ and CD4+ cells during the REP. Figure S5: Phenotyping of young TILs and REP TILs in Patient 05. Figure S6: Flow‐cytometry based phenotyping of TIL infusion products. Figure S7: Tumor reactivity of TILs from Patient 01. Figure S8: Tumor reactivity of TILs from Patients 05 and 06. Table S1: Staining panels used for flow‐cytometry based phenotyping of TIL infusion products. [file IJC-159-1735-s001.pdf]

Supplementary material

Tumor infiltrating lymphocyte therapy combined with PD-1/LAG-3 inhibition in patients with recurrent platinum-resistant ovarian cancer

Tine J. Monberg, Cathrine L. Lorentzen, Marie C. W. Westergaard, Trine Z. Iversen, Troels H. Borch, Marco Donia, Sigrid M. Mannering, Stine E.W. Banke, Özcan Met, Inge Marie Svane

Table of content

Paragraph S1: Inclusion and exclusion criteria 2

Figure S1. TIL expansion and reactivity testing workflow 4

Table S1: Staining panels used for flow-cytometry based phenotyping of TIL infusion products 5

Figure S2. Individual changes in CA-125 and tumor burden during treatment 6

Figure S3 Treatment efficacy. 7

Figure S4. Changes in populations of CD8+ and CD4+ cells during the REP 8

Figure S5. Phenotyping of young TILs and REP TILs in patient 05. 9

Figure S6. Flow-cytometry based phenotyping of TIL infusion products. 10

Figure S7. Tumor reactivity of TILs from patient 01 12

Figure S8. Tumor reactivity of TILs from patients 05 and 06. 13

Paragraph S1: Inclusion and exclusion criteria

Inclusion Criteria

All the criteria listed in the following need to be met before patient inclusion.

1. Histological proven advanced ovarian-, fallopian tube or primary peritoneal cancer with the possibility of surgical removal of tumor tissue of > 1 cm3. All histologies can be included.
2. Progressive or recurrent resistant disease after platin-based chemotherapy (platinum resistant) or progressive or recurrent disease after second line or additional chemotherapy.
3. Age: 18 – 75 years.
4. ECOG performance status of ≤1
5. Life expectancy of > 6 months.
6. At least one measurable parameter in accordance with RECIST 1.1 criteria.
7. LVEF assessment with documented LVEF ≥50% by either TTE or MUGA (TTE preferred test) within 6 months from first study drug administration
8. No significant toxicities or side effects (CTC ≤ 1) from previous treatments, except sensory- and motoric neuropathy (CTC ≤ 2) and/or alopecia (CTC ≤ 2).
9. Sufficient organ function, including:
   - Absolute neutrophil count (ANC) ≥ 1.500 /µl
   - Leucocyte count ≥ lower normal limit
   - Platelets ≥ 100.000 /µl and <700.000 /µl
   - Hemoglobin ≥ 6,0 mmol/l (regardless of prior transfusion)
   - S-creatinine < 140
   - S-bilirubin ≤ 1,5 times upper normal limit
   - ASAT/ALAT ≤ 2,5 times upper normal limit
   - Alkaline phosphatase ≤ 5 times upper normal limit
   - Lactate dehydrogenase ≤ 5 times upper normal limit
   - Sufficient coagulation: APPT<40 and INR<1,5
10. Signed statement of consent after receiving oral and written study information
11. Willingness to participate in the planned controls and capable of handling toxicities.
12. Age and Reproductive Status: Females, ages ≥18 years, inclusive:
    - Women of childbearing potential (WOCBP) must have a negative serum or urine pregnancy test (minimum sensitivity 25 IU/L or equivalent units of human chorionic gonadotropin (hCG)) within 24 hours prior to the start of study treatment. Women of childbearing potential (WOCBP) must agree to follow instructions for method(s) of contraception. This applies from inclusion in the study and for the duration of treatment with Ipilimumab, Relatlimab and Nivolumab plus 5 half-lives of study treatment plus 30 days (duration of ovulatory cycle) for a total of 24 weeks post-treatment completion. The following are considered safe methods of contraception: Hormonal anticonception (birth control pills, spiral, depot injection with gestagen, subdermal implantation, hormonal vaginal ring and transdermal depot patch), intrauterine device, surgical sterilization, surgical sterilization of male partner with verification of no sperm after the procedure, menopause (for more than 12 months)

Exclusion criteria

Patients will be excluded if they meet one of the criteria's listed below

1. A history of prior malignancies. Patients treated for another malignancy can participate if they are without signs of disease for a minimum of 3 years after treatment.
2. Known hypersensitivity to one of the active drugs or one or more of the excipients.
3. Severe medical conditions, such as severe asthma/COLD, significant cardiac disease, poorly regulated insulin dependent diabetes mellitus among others.
4. Creatinine clearence < 70 ml/min*.
5. Acute/chronic infection with HIV, hepatitis, syphilis among others.
6. Severe allergies or previous anaphylactic reactions.

7. Active autoimmune disease, such as autoimmune neutropenia/thrombocytopenia or hemolytic anemia, systemic lupus erythematosis, Sjögren's syndrome, sclerodermia, myasthenia gravis, Goodpasteur's disease, Addison's disease, Hashimotos thyroiditis, active Graves disease.
8. Subjects with history of myocarditis, regardless of etiology
9. Troponin T (TnT) or I (TnI) > 2x institutional upper limit of normal (ULN) is excluded. ii) between > 1 to 2 x ULN will be permitted if a repeat assessment remains ≤ 2 x ULN and participant undergoes a cardiac evaluation and is cleared by a cardiologist or cardio-oncologist
10. Prior treatment with LAG-3 targeted agents.
11. Pregnant women and women breastfeeding.
12. Simultaneous treatment with systemic immunosuppressive drugs (including prednisolone, methotrexate among others)**.
13. Simultaneous treatment with other experimental drugs. Based on clinical judgement anti-hormonal treatment can be accepted.
14. Simultaneous treatment with other systemic anti-cancer treatments.
15. Patients with active and uncontrollable hypercalcemia

* In selected cases it can be decided to include a patient with a GFR < 70 ml/min with the use of a reduced dose of chemotherapy.
** In selected cases a systemic dose of ≤10 mg prednisolone or a transient planned treatment that can be stopped before TIL therapy can be tolerated.

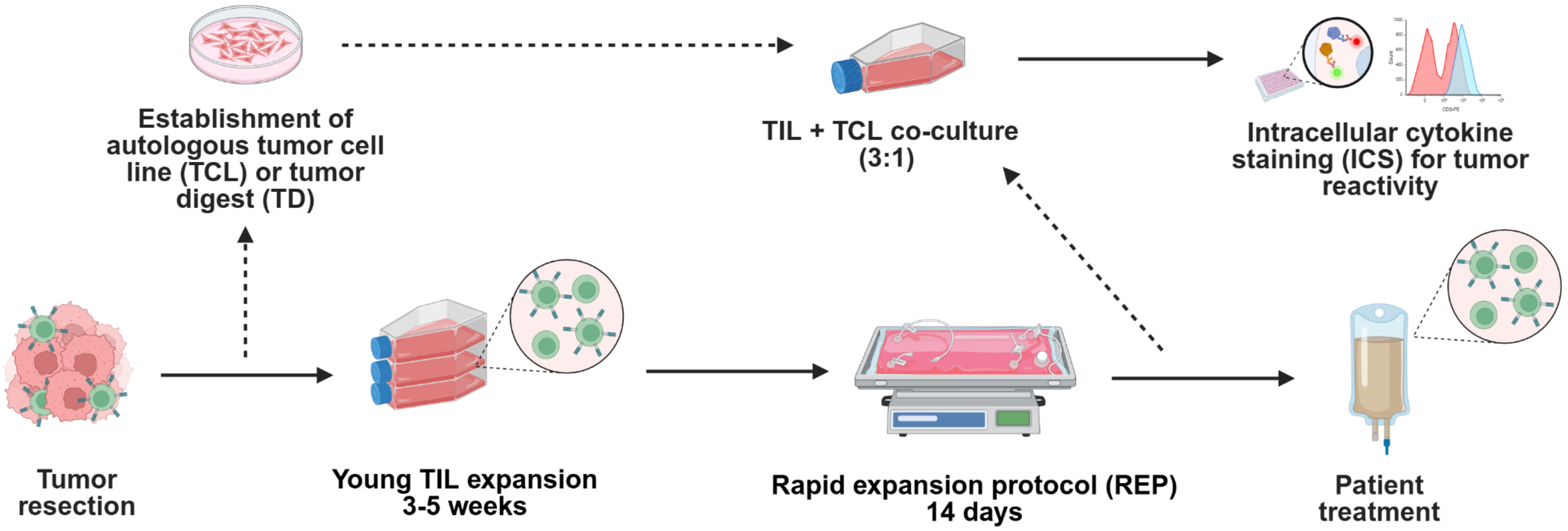

Figure S1. TIL expansion and reactivity testing workflow. Young TILs derived from tumor fragment were expanded for 3-5 weeks and subsequently transferred to a bioreactor for further expansion in accordance with the rapid expansion protocol (REP). The resulting TIL infusion product was administered to the patient preceded by 7 days of lymphodepleting chemotherapy. For reactivity testing an autologous tumor cell line (TCL) or tumor digest (TD) was established from the resected tumor tissue. TCL or TD was co-cultured with REP-expanded TILs and reactivity was assessed by flow-cytometry based intracellular cytokine staining (ICS). Created with Biorender.com.

Panel 1	Antigen	Panel 2	Antigen	Panel 3	Antigen
FITC	**CD45**	FITC	**CD57**	FITC	**LAG3**
PerCP-Cy5.5 or BB700		PerCP-Cy5.5 or BB700	**HLA-DR**	PerCP-Cy5.5 or BB700	
APC	**TCRab**	APC	**CD28**	APC	**BTLA**
APC-R700	**CD8**	APC-R700	**CD8**	APC-R700	**CD8**
APC-Cy7	**NIR**	APC-Cy7	**NIR**	APC-Cy7	**NiR**
BV421		BV421	**CD45RO**	BV421	**TIGIT**
BV510	**CD4**	BV510	**CD4**	BV510	**CD4**
BV605	**CD56**	BV605		BV605	
BV650		BV650	**CD45RA**	BV650	**CD69**
BV711	**TCRgd**	BV711	**CD27**	BV711	**TIM3**
BV786		BV786	**CD3**	BV786	**CD3**
PE	**CD3**	PE	**CCR7**	PE	**CD103**
PE-CF594/Da		PE-CF594/da	**PD-1**	PE-CF594/da	**PD-1**
PE-AF700		PE-AF700		PE-AF700	**CD29**
PE-Cy7	**CD19**	PE-Cy7	**CD95**	PE-Cy7	**CD39**

Table S1: Staining panels used for flow-cytometry based phenotyping of TIL infusion products

(A)

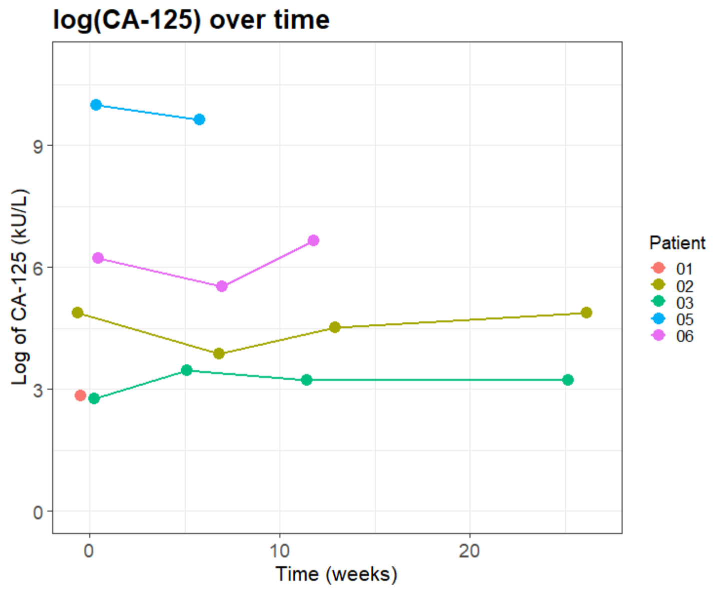

(B)

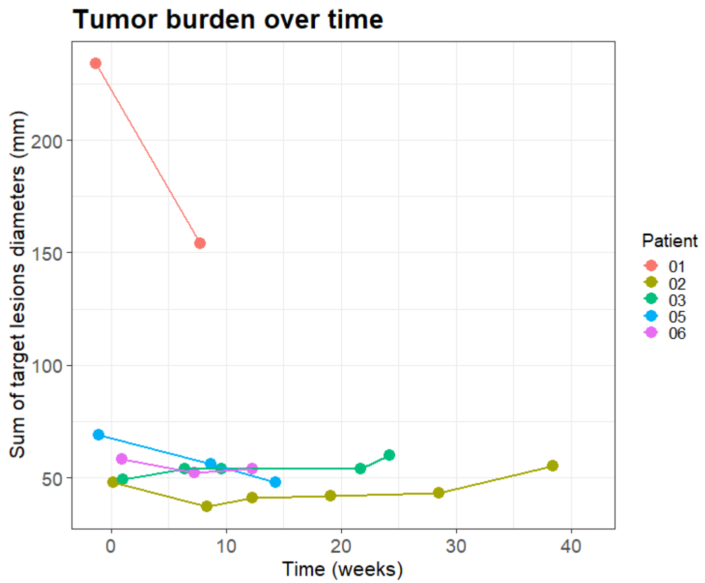

CA 125 (kU/L)

PATIENT	BASELINE	6 WEEKS	12 WEEKS	6 MONTHS
01	17	-	-	-
02	130	48	92	130
03	16	32	25	25
05	21700	15420	-	-
06	500	250	769	-

Figure S2. Individual changes in CA-125 and tumor burden during treatment. (A) CA-125 changes are shown as log-transformed values and absolute values are presented in the table. **(B)** Changes in the sum of target lesions diameter (mm) during treatment. Two patients (01 and 05) achieved a reduction in tumor burden consistent with a partial response according to RECIST 1.1.

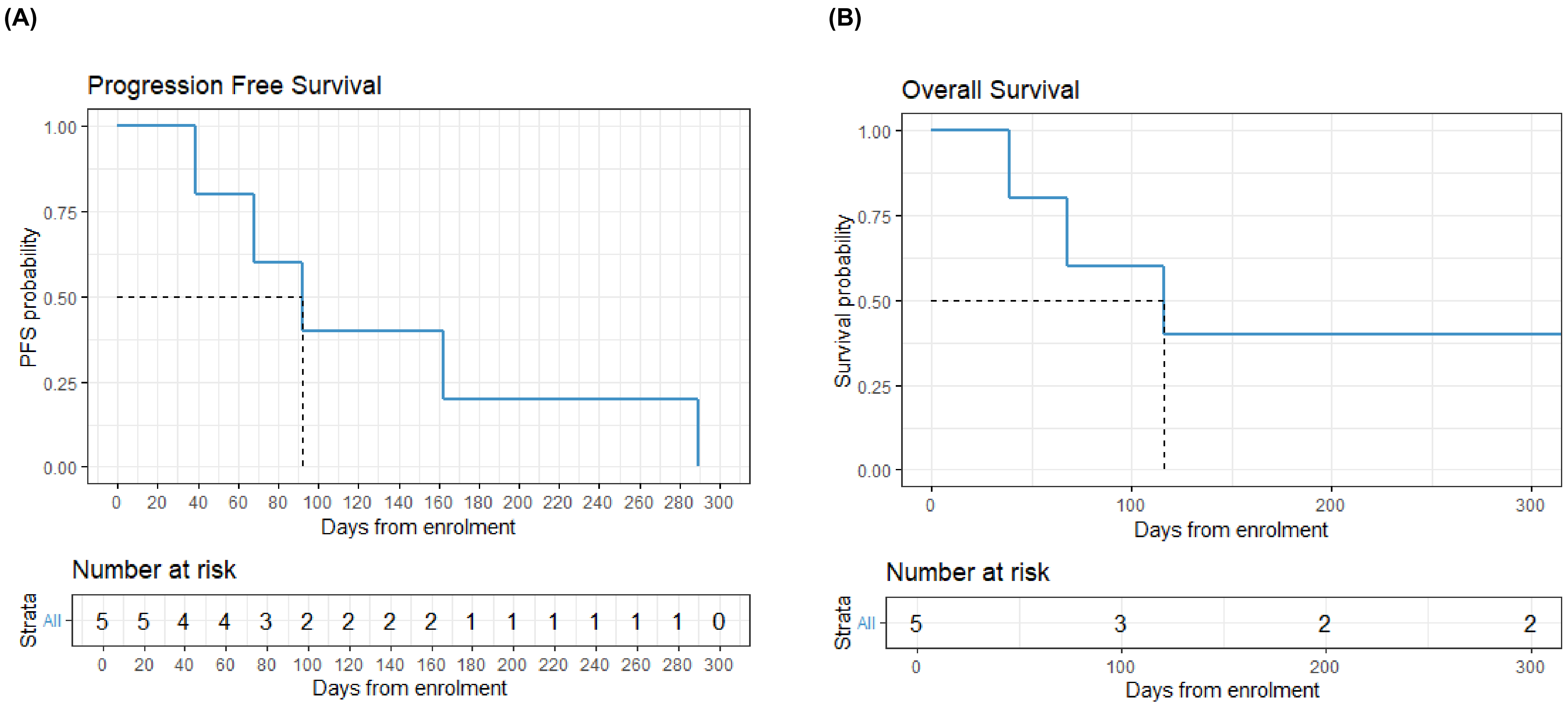

Figure S3 Treatment efficacy. (A) Progression Free survival (PFS) illustrated as time to progression or death from any cause, medianPFS = 92 days. **(B)** Overall survival. Median OS = 116 days.

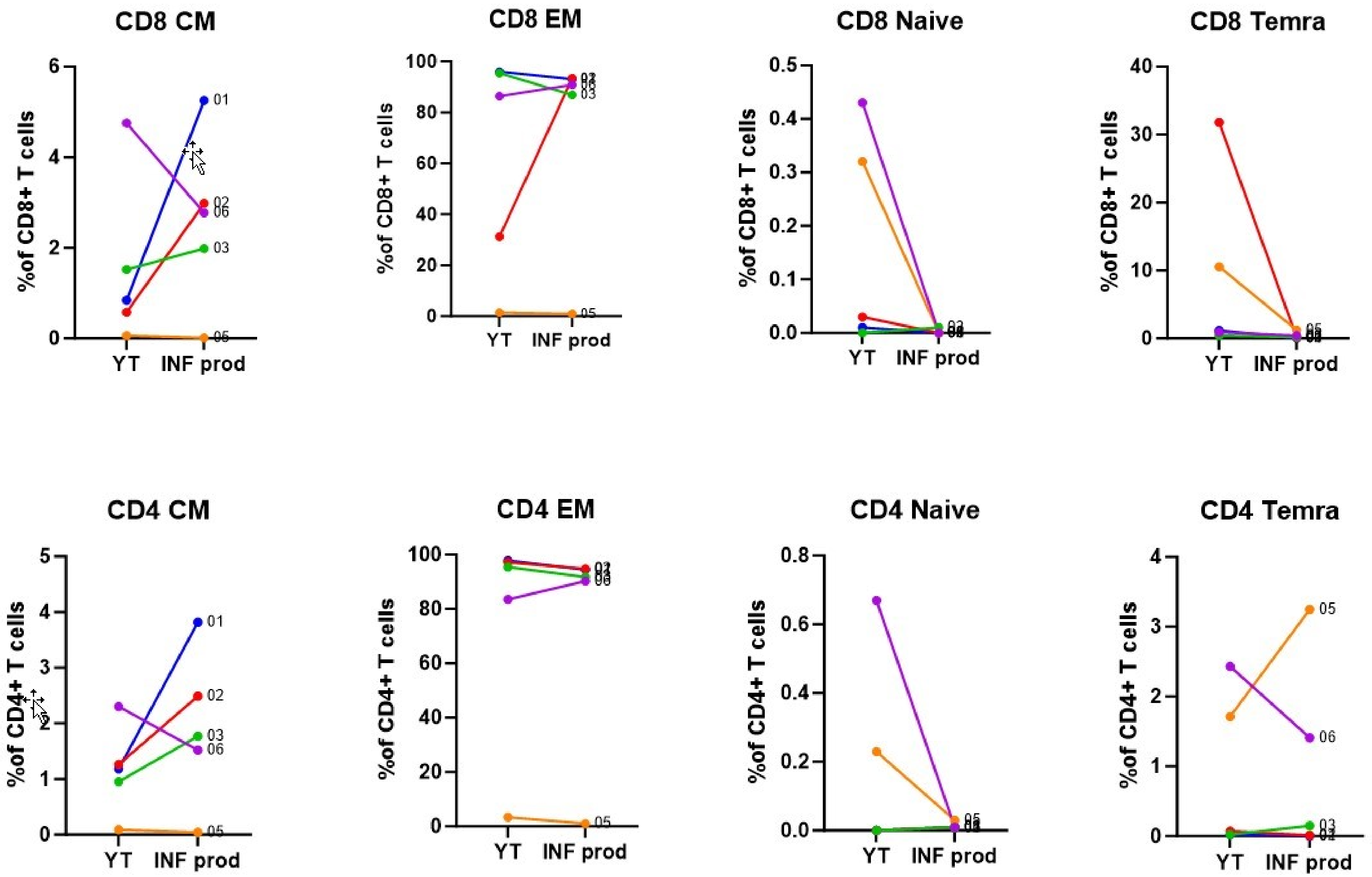

Figure S4. Changes in populations of CD8+ and CD4+ cells during the REP. No clear tendencies were observed. CM = central memory, EM = Effector memory, TEMRA = terminally differentiated effector memory T-cells.

Young TILs, CD4+

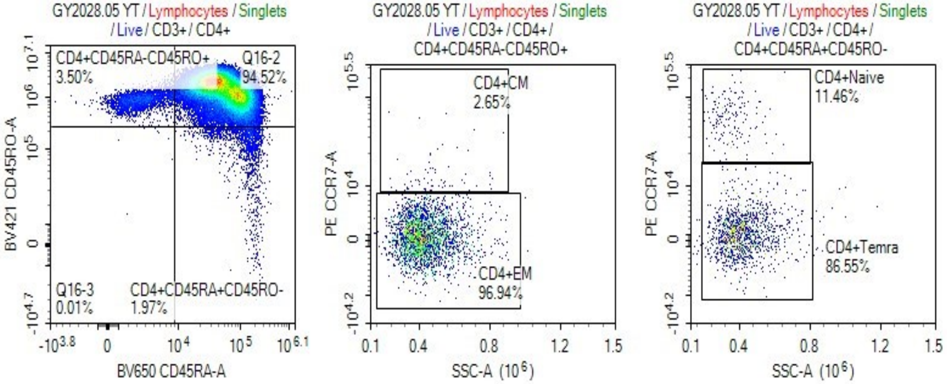

Young TILs, CD8+

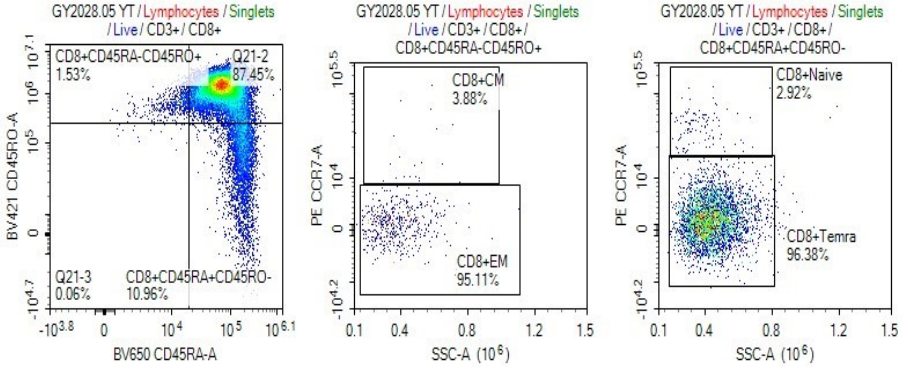

REP TILS, CD4+

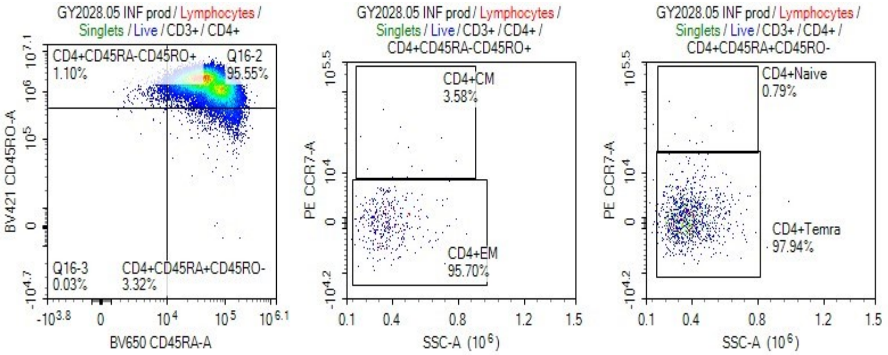

REP TILS, CD8+

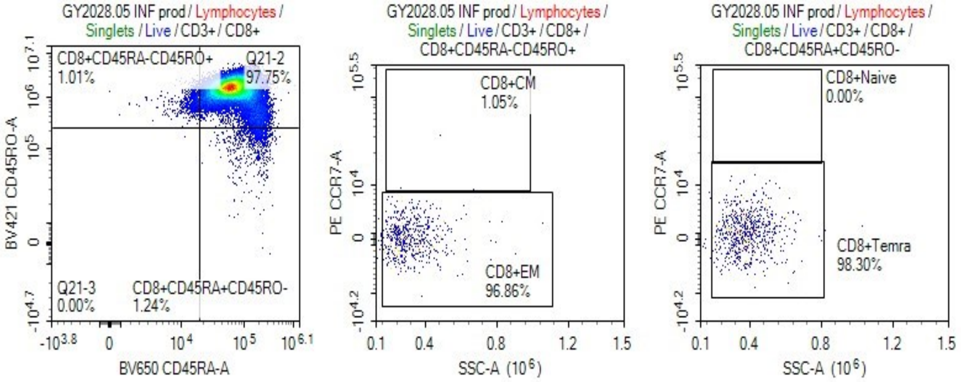

Figure S5. Phenotyping of young TILs and REP TILs in patient 05. In both young TILs and infusion product the CD4+ and CD8+ T cell populations were dominated by CD45RA+CD45RO+ T cells. These cells were further characterized as negative for CCR7. REP, rapid expansion protocol. TILs, tumor infiltrating lymphocytes.

(A)

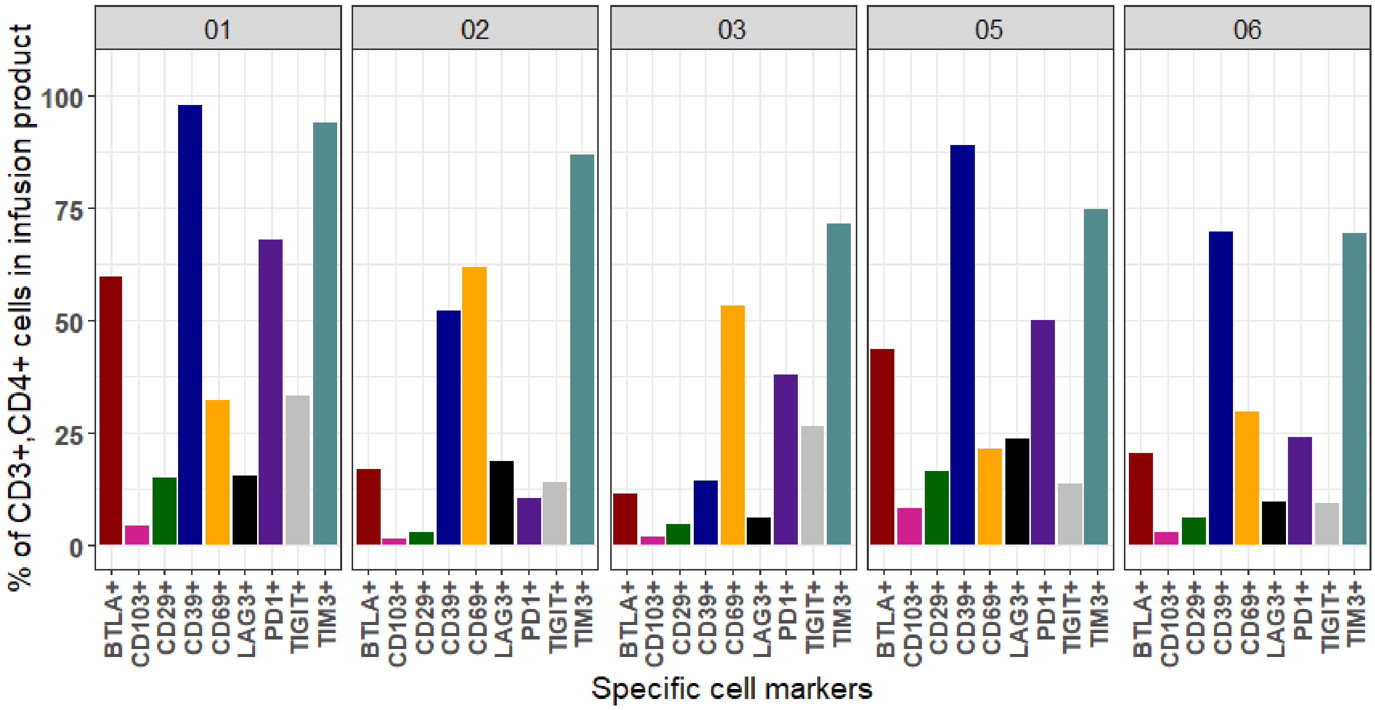

(B)

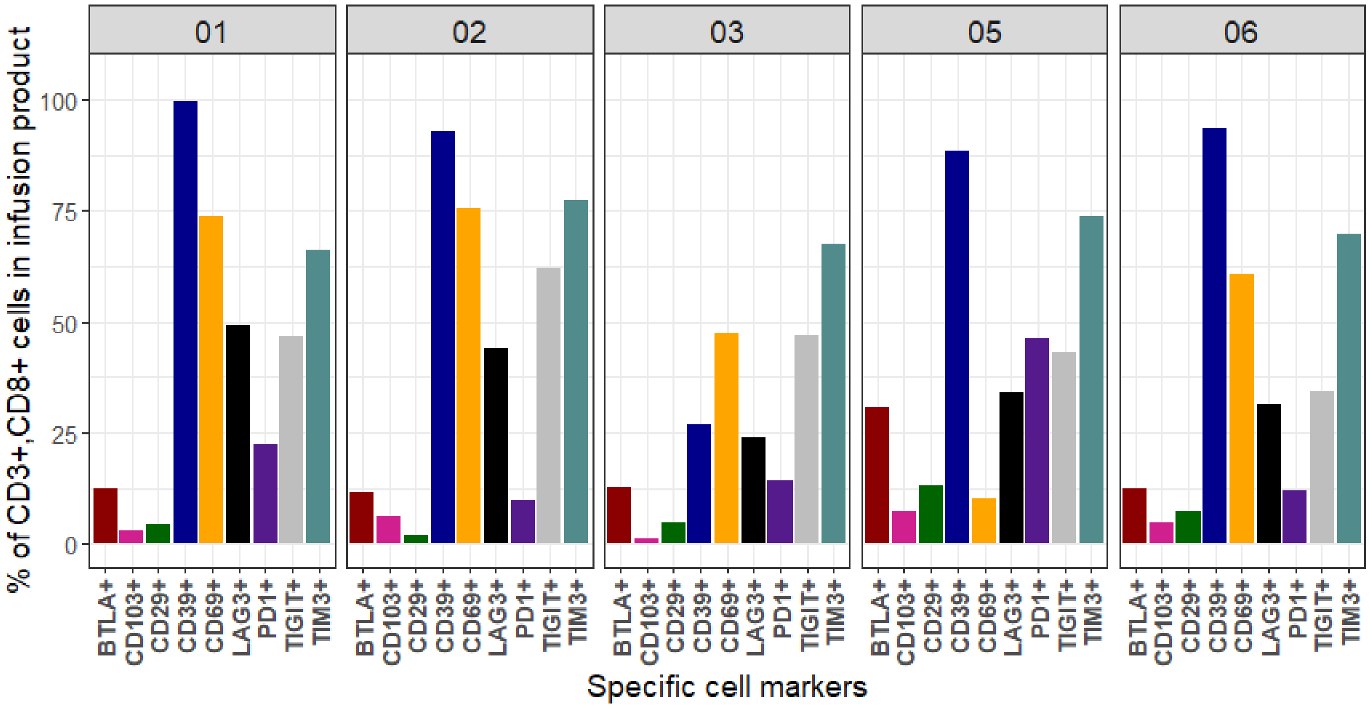

Figure S6. Flow-cytometry based phenotyping of TIL infusion products. Expression of selected markers (panel 3, supplementary table S1) in the subsets of **(A)** CD4+ cells and **(B)** CD8+ cells. Except for high CD39 expression and a low CD103 expression, no clear patterns were observed.

(A)

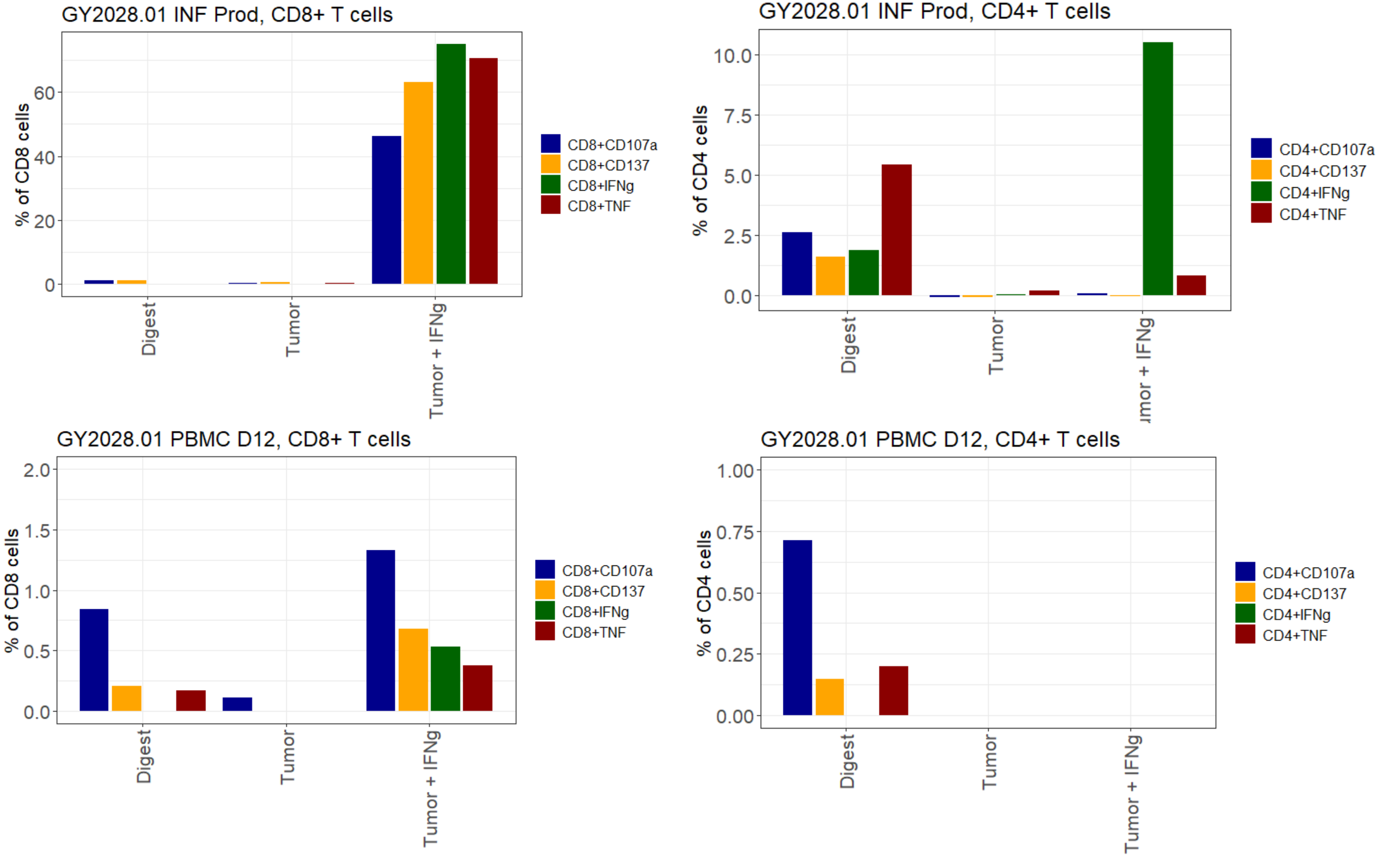

GY2028.01 INF Prod, CD8+ T cells
% of CD8 cells
0
20
40
60
Digest
Tumor
Tumor + IFNg
CD8+CD107a
CD8+CD137
CD8+IFNg
CD8+TNF
GY2028.01 INF Prod, CD4+ T cells
% of CD4 cells
0.0
2.5
5.0
7.5
10.0
Digest
Tumor
ımor + IFNg
CD4+CD107a
CD4+CD137
CD4+IFNg
CD4+TNF
GY2028.01 PBMC D12, CD8+ T cells
% of CD8 cells
0.0
0.5
1.0
1.5
2.0
Digest
Tumor
Tumor + IFNg
CD8+CD107a
CD8+CD137
CD8+IFNg
CD8+TNF
GY2028.01 PBMC D12, CD4+ T cells
% of CD4 cells
0.00
0.25
0.50
0.75
1.00
Digest
Tumor
Tumor + IFNg
CD4+CD107a
CD4+CD137
CD4+IFNg
CD4+TNF

(B)

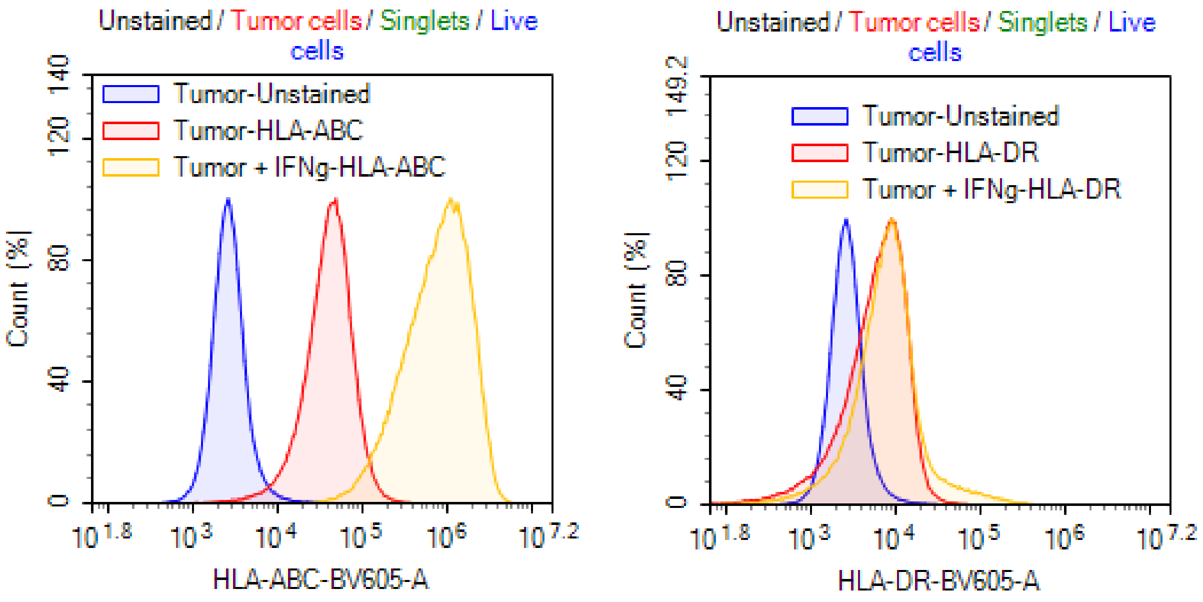

	Median MFI HLA-ABC	HLA-DR
Tumor US	2673	2673
Tumor	42378	6355
Tumor +IFNg US	2935	2935
Tumor +IFNg	837761	7912
**Minus background**		
Tumor	39705	3682
Tumor +IFNg	834826	4977
**Fold increase**	**21.0**	**1.4**

Figure S7. Tumor reactivity of TILs from patient 01. (A) Reactivity of TIL infusions product and post-infusion peripheral blood mononuclear cells (PBMCs) towards autologous tumor cells lines and/or tumor digest in patient 01. Reactivity testing was performed using intracellular cytokine staining. **(B)** Major histocompatibility complex (MHC) I and II expression on tumor cells from patient 01 before and after stimulation with interferon gamme (IFNg). The expression is shown as median fluorescence intensity (MFI). Upon IFNg stimulation, the expression of MHC I (HLA-ABC) increased by 21-fold, while the expression of MHC II (HLA-DR) was almost unaffected.

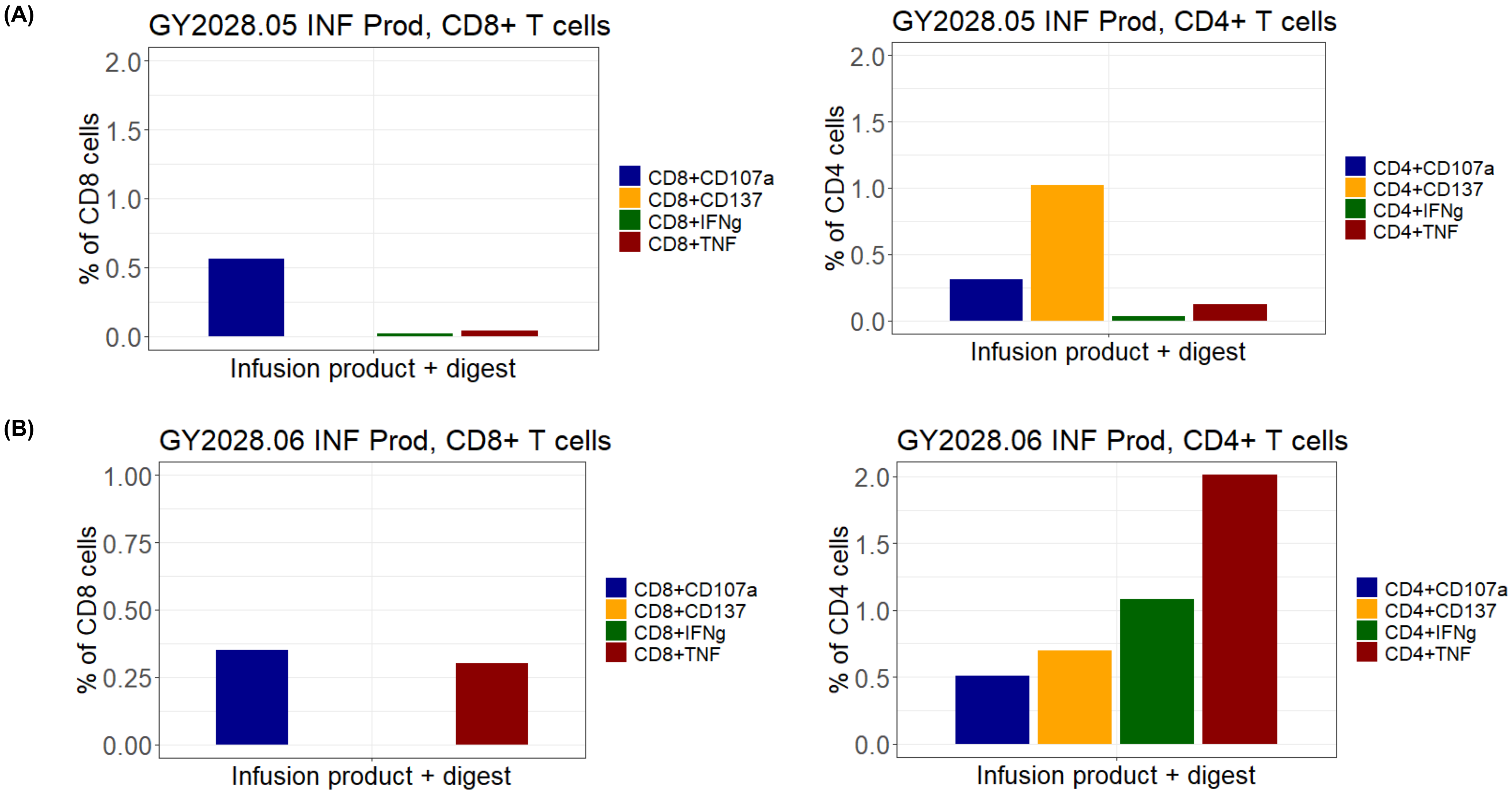

Figure S8. Tumor reactivity of TILs from patients 05 and 06. Reactivity of CD8+ and CD4+, respectively, in infusion products towards tumor digest in patients 05 **(A)** and 06 **(B).** Reactivity testing was performed using intracellular cytokine staining.
